# Supplementary material for: The effects of DMSO on DNA conformations and mechanics
Source: Biophys J. 2025 Jul 2;124(16):2639–54. doi: 10.1016/j.bpj.2025.06.041 (PMC12414676; doi:10.1016/j.bpj.2025.06.041)
Supplement: Supplementary file 2 [file mmc3.pdf]

Biophysical Journal
Article

The effects of DMSO on DNA conformations and mechanics

Koen R. Storm,[1,4] Caroline Körösy,[1] Enrico Skoruppa,[2] Stefanie D. Pritzl,[1] Pauline J. Kolbeck,[1] Willem Vanderlinden,[3] Helmut Schiessel,[2] and Jan Lipfert[1,4,*]

[1]Soft Condensed Matter and Biophysics, Department of Physics and Debye Institute for Nanomaterials Science, Utrecht University, Utrecht, the Netherlands; [2]Cluster of Excellence Physics of Life, TU Dresden, Dresden, Germany; [3]School of Physics and Astronomy, University of Edinburgh, Scotland, United Kingdom; and [4]Institute for Physics, University of Augsburg, Universitätsstrasse 1, Augsburg, Germany

ABSTRACT Dimethyl sulfoxide (DMSO) is a polar aprotic solvent used in a wide range of applications, including uses as a drug and in drug delivery, as a solvent for fluorescent dyes, and in enzymatic reactions that process DNA. Consequently, many assays contain low concentrations (≤10%) of DMSO. While it is well known that DMSO lowers the melting temperature of DNA, its effects on DNA conformations and mechanical properties below the melting temperature are unclear. Here, we use complementary single-molecule techniques to probe DNA in the presence of 0–60% DMSO. Magnetic tweezers force-extension measurements find that the bending persistence length of DNA decreases moderately and linearly with DMSO concentrations up to 20 vol %, by (0.43 ± 0.02%) per %-DMSO. Magnetic tweezers twist measurements demonstrate a reduction in melting torque in the presence of DMSO and find that the helical twist of DNA remains largely unchanged up to 20% DMSO, while even higher concentrations slightly unwind the helix. Using AFM imaging, we find a moderate compaction of DNA conformations by DMSO and observe a systematic decrease of the mean-squared end-to-end distance by 1.2% per %-DMSO. We use coarse-grained Monte Carlo simulations of DNA as a semiflexible polymer with a variable density of flexible segments, representing DMSO-induced local defects or melting, to rationalize the observed behavior. The model quantitates the effects of introducing locally flexible regions into DNA and gives trends in line with the magnetic tweezers and AFM imaging experiments. Our results show that addition of up to 50% DMSO has a gradual effect on DNA structure and mechanics and that, for low concentrations (≤20%), the induced changes are relatively minor. Our work provides a baseline to understand and model the effects of DMSO on DNA in a range of biophysical and biochemical assays.

SIGNIFICANCE Dimethyl sulfoxide (DMSO) is a widely used polar aprotic solvent. It is employed, e.g., as a drug and in drug delivery, as a solvent for small molecules, and as an additive to enzymatic reactions that process DNA. Despite its widespread use, its effects on DNA conformations and properties are not well understood and often neglected. We use single-molecule manipulation with magnetic tweezers and AFM imaging to probe DNA in the presence of 0–60% DMSO. We find that DMSO increases the flexibility of DNA, leading to more compact conformations. Up to 50% DMSO, the induced changes are gradual and approximately linear. Our results provide a quantitative baseline to understand, model, and optimize assays with DNA in the presence of DMSO.

INTRODUCTION

Dimethyl sulfoxide (DMSO) is a polar aprotic solvent and widely used in a large range of applications (1). It is used clinically to treat interstitial cystitis (2–4) and acts as a topical analgesic, anti-inflammatory, and antioxidant (5,6). DMSO is frequently used as a solvent for stock solutions of, e.g., small molecule drugs and fluorescent dyes. As a result, many biological and biochemical assays contain DMSO in low concentration as an inadvertent component (7,8). In addition, DMSO is employed as a cryo- and radioprotectant (9–11), and frequently used as an additive to increase yield in biochemical reactions, in particular in PCR, transcription, and topoisomerization assays (12–21). DMSO is well known to lower the melting temperature of DNA (22,23); however, it is less clear how it affects overall DNA structure and mechanical properties (24–26). While the effects of low concentrations of DMSO in aqueous buffers on DNA mechanics are often neglected, some

Submitted March 11, 2025, and accepted for publication June 30, 2025.

*Correspondence: j.lipfert@uu.nl

Koen R. Storm and Caroline Körösy contributed equally to this work.

Editor: Lars Nordenskiold.

https://doi.org/10.1016/j.bpj.2025.06.041

© 2025 The Authors. Published by Elsevier Inc. on behalf of Biophysical Society.
This is an open access article under the CC BY license (http://creativecommons.org/licenses/by/4.0/).

studies suggest that they can induce significant changes of DNA stiffness. For example, recent work using atomic force microscopy (AFM) imaging reported an approximately fourfold reduction in DNA bending stiffness in 3% DMSO (27).

In general, the solution environment and cosolutes can strongly influence the properties of DNA, in particular its stiffness (28–31). Since DNA is highly negatively charged, its properties are affected by the ionic strength of the solution (30–37). Specifically, increasing salt concentrations lead to a decrease in bending stiffness (but not torsional stiffness (38)), since the DNA-DNA interactions are screened (31,33,39–41). However, the salt dependence of the bending stiffness is most pronounced below physiological salt concentrations (<100 mM monovalent salt). Organic solvents and cosolutes can have a range of effects on DNA (24,42–46). For example, polyols including glycerol have been shown to destabilize the DNA helix and lower the melting temperature, due to specific interactions with the bases (47), similar to what has been proposed for DMSO.

In addition to specific interactions, many organic cosolutes will lower the dielectric constant of the solution (which is large, $\varepsilon \approx 80$, for water) (24). Lowering the dielectric constant increases all electrostatic interactions, including DNA-DNA interactions (which would increase the DNA stiffness and lower its stability), but also DNA-ion interactions (which tend to stabilize the duplex and decrease its bending stiffness) as well as ion-ion, ion-water, etc., interactions. The result is a complicated interplay of compensatory effects. For example, ethanol ($\varepsilon \approx 24$) has been shown to decrease the bending stiffness of DNA, which has been suggested to result from an increase in ion association (48).

Here, we use single-molecule assays to directly probe the effects of DMSO on the overall conformations, bending stiffness, and helical twist of DNA. In a first set of measurements, we use magnetic tweezers (MT) to determine the stretching and twist response of DNA in the presence of varying concentrations of DMSO, ranging from 0 to 60% DMSO (v/v) in a background of (near-)physiological phosphate-buffered saline (PBS) buffer. In MT, DNA molecules are tethered between a flow cell surface and $\sim$1 μm diameter superparamagnetic beads (49,50). External magnets enable the application of precisely calibrated stretching forces (51) and control of the DNA linking number (29,49). From force-extension measurements, we find a moderate and initially linear decrease of the bending persistence length with increasing DMSO concentration, by (0.43 $\pm$ 0.02%) per %-DMSO. From extension-rotation measurements at constant applied force, we observe essentially no change in DNA twist, except for the highest DMSO concentrations, but a reduction of the melting torque upon addition of DMSO.

In a second set of experiments, we use AFM imaging of linear DNA in air to directly observe the conformations of free double-stranded DNA (dsDNA) in the presence of varying concentrations of DMSO in the absence of applied forces and torques (52–55). We observe a moderate compaction of DNA upon addition of DMSO and a corresponding average reduction of the end-to-end distance, by 1.2% per %-DMSO.

To rationalize the observations from our single-molecule measurements in a simple, but quantitative model, we perform extensive coarse-grained Monte Carlo simulations of dsDNA, both with applied external forces and in free solution. We consider our coarse-grained simulations with implicit solvent as a minimal model. The simulations do not treat individual bases and do not provide direct insights into the interactions of DMSO with DNA bases, but they have the advantage of enabling simulations of DNA molecules in the length range used experimentally ($\sim$kbp) (56–62) and to test different scenarios and parametrizations of the effects of DMSO, which is critical in the absence of a clear a priori knowledge of its molecular effects. We model the effect of DMSO by adding flexible defect segments or "bubbles," i.e., DNA positions that are much more flexible than the regular segments and thus can accommodate local bending more readily than standard B-form DNA. In the simulations, we systematically vary bubble size, number, and stiffness. Our simulations do not include explicit electrostatics, which is a limitation, but we note that DMSO still has a relatively large dielectric constant ($\varepsilon \approx 47$ for pure DMSO) and that an accurate treatment of electrostatics would likely require quantitatively capturing subtle changes in DNA-DNA, DNA-ion, and DNA-DMSO interactions with DMSO concentration, which would require finer-grained simulations. We find that an increasing density of bubbles leads to a decrease of the apparent persistence length and end-to-end lengths, consistent with the experimental observations for DNA in the presence of DMSO and in agreement with the notion that the presence of kinks renormalizes the bending persistence length (63,64).

MATERIALS AND METHODS

Chemicals and preparation of dilution series

DMSO was purchased from Sigma-Aldrich (DMSO, for molecular biology [$\geq$99.9%]; catalog number D8418, Sigma-Aldrich,Burlington, MA, USA). The DMSO solutions were prepared in 1$\times$ PBS (consisting of 10 mM phosphate buffer [pH 7.4], with 137 mM NaCl and 2.7 mM KCl; catalog number P3813, Sigma-Aldrich) for concentrations of 1–60% (v/v) DMSO. Refractive indices of the solutions were determined using an Abbe refractometer (Abbe Refractometer 3T, ATAGO, Tokyo, Japan; at λ = 589.3 nm and 21°C) for MT analysis (Fig. S1).

DNA construct and flow cell preparation for MT measurements

For MT measurements, we used a functionalized 20.6 kbp DNA construct that was described previously (65). The DNA construct has ($\approx$600 bp) PCR-generated handles with multiple biotin or digoxigenin groups ligated

to the ends of the unmodified central sequence of the DNA. Streptavidin-coated, 1.05 μm diameter superparamagnetic beads (MyOne, Thermo Fisher Scientific, Waltham, MA, USA) were attached to the DNA constructs by binding to the biotin moieties, by adding 1 μL of picomolar DNA stock solution to 2 μL of the magnetic beads in 200 μL 1× PBS.

Flow cells were prepared using two microscope coverslips (24 × 60 mm; Carl Roth, Karlsruhe, Germany). The bottom coverslip was functionalized with (3-glycidoxypropyl)trimethoxysilane and incubated with 100 μL of a 5000× diluted stock solution of 1 μm diameter polystyrene beads (Polysciences, Warrington, PA, USA) in ethanol, to be used as reference beads. Alternatively, for some measurements, unspecifically stuck MyOne superparamagnetic beads were used as reference beads. For liquid exchange in the flow cell, the top coverslip was prepared with two openings with a 1 mm radius. Flow cells were assembled by connecting the two coverslips using a layer of parafilm (Carl Roth), with a central 50 μL channel cut out, and sealed by heating on a hot plate at 100°C for approximately 20 s.

After flow cell assembly, 100 μL of 100 μg/mL anti-digoxigenin (Abcam, Cambridge, UK) in 1× PBS were introduced and incubated for at least 1 h. The flow cell was passivated using 100 μL of 250 mg/mL bovine serum albumin (Carl Roth) for 1 h to counter nonspecific interactions. The bead-coupled DNA constructs were introduced into the flow cell and allowed to bind via multiple digoxigenin:anti-digoxigenin interactions to the bottom coverslip during a 30 min incubation.

MT setup

A custom-built single-molecule MT setup as described previously was used to perform experiments on DNA (66,67). The setup employs two 5 × 5 × 5 mm^3 permanent magnets (W-05-N50-G; Supermagnete) with a 1 mm gap placed in a vertical configuration (51). A DC translational motor (M-126.PD2, Physik Instrumente (PI), Karlsruhe, Germany) was used to control the distance between magnets and the flow cell, together with a rotational motor (C-150.PD, PI) to control the rotation of the magnets. We used a LED (69,647; LUMITRONIX LED Technik, Hechingen, Germany) for illumination. A 40× oil-immersion objective (UPLFLN 40×; Olympus, Tokyo, Japan) and a CMOS sensor camera with 4096 × 3072 pixels (12M Falcon2; Teledyne DALSA, Waterloo, Ontario, Canada) were used to image a field of view of 400 × 300 μm^2. Images recorded at 58 Hz were transferred to a frame grabber (PCIe 1433; National Instruments, Austin, TX, USA). The images were tracked in real time using custom tracking software (Labview, National Instruments) to extract the beads' (x, y, z) coordinates (68,69). The objective was positioned on a piezo stage (PifocP726.1CD, Physik Instrumente) to create a look-up table for tracking of the beads' z-position. The look-up table was generated over a range of 20 μm, with a step size of 100 nm. Labview routines described previously were used for setup control and bead tracking (69). For measurements at different DMSO concentrations, the tracked positions using the look-up table were corrected for the change in index of refraction with DMSO concentration (Fig. S1).

MT measurements

Before measuring, the DNA tethered beads are subjected to force and torque to detect the binding to multiple tethers and to test for torsional constraint. First, negative turns are applied under high tension (F = 5 pN). At a high tension, rather than buckling to form plectonemes, melting of DNA occurs upon applying negative turns (70) (corresponding to underwinding of the DNA), preventing a decrease in extension. However, in the case of multiple dsDNA tethers attached to the same bead, a decrease in extension is observed as the tethers braid upon rotation of the magnetic bead. Beads bound to multiple tethers are not analyzed further. To test for breaks or nicks in the DNA tether, positive turns are applied at low tension (F = 0.5 pN). In the case of torsionally constrained DNA, the tethers will overwind and form plectonemes, consequently decreasing the extension. In contrast, torsionally unconstrained tethers will not overwind and no plectonemes are formed.

First, rotation-extension measurements were performed on torsionally constrained tethers at several forces (0.25, 0.5, 0.8, and 1 pN) by determining the extension while varying the relative turns of the magnet. The linking number was changed in steps of five turns at 0.25 pN and in steps of 10 turns at 0.5 pN and higher; at each force and number of applied turns, the extension was measured for 6 s and averaged. To determine the initial orientation of the tethers, a rotation curve was measured in 1× PBS. While applying a constant force, the magnets are rotated to positive or negative turns, and the extension is monitored. From this, the relative number of turns to achieve maximum extension and thus fully torsionally relaxed tethers (which we designate as zero turns) is determined. Second, the force-extension response was determined by measuring the tether extension while placing the magnets subsequently at 11 positions corresponding to forces between 0.07 and 5 pN.

The effects of different concentrations of DMSO on the rotation-extension and force-extension behavior of DNA were investigated by subsequently changing DMSO concentration and repeating the rotation and force-extension measurements. At each concentration step, 200–300 μL (corresponding to 4–6 flow cell volumes) of DMSO solution at defined concentrations was introduced into the flow cell using a peristaltic pump, in order from low to high concentration, unless otherwise noted. We applied a force of 2 pN while flushing to constrain movement of the bead and to prevent changes in bead rotation during fluid exchange.

DNA preparation for AFM imaging

Linear dsDNA was obtained by cutting the commercially available DNA vector pBR322 (New England Biolabs [NEB], Ipswich, MA, USA) with the FastDigest enzyme *Eco*RI (Thermo Fisher Scientific). Following the manufacturer's instructions, we scaled up the reaction using 5 μg of plasmid DNA in a total volume of 50 μL. The enzymatic reaction was stopped by heat inactivation and the success of the digest was confirmed in a 1% agarose gel using ROTI-Gel stain (Carl Roth) for DNA visualization and 1 kb Plus DNA Ladder (NEB) as a size reference (Fig. S2). The linearized DNA was purified using a PCR clean up kit (QIAGEN, Germantown, MD, USA). Differing from the manufacturer's clean up protocol, the DNA was washed in three steps and eluted in two steps of 25 μL 1× PBS after an incubation time of 1 min at 37°C.

AFM imaging

For imaging we use a Nanowizard Ultraspeed 2 high speed AFM (JPK, Berlin, Germany) with triangular FASTSCAN A probes (Bruker, Billerica, MA, USA) with a driving frequency of ~1400 kHz and a spring constant of 18 N/m. Overview images for analysis were scanned in tapping mode in air over a field of view of 6 × 6 μm^2 at 512 × 512 pixels and a line scanning speed of 1 Hz. High-resolution images for additional visualization were recorded with 2048 × 2048 pixels in a field of view 1 × 1 μm^2. Data for end-to-end distances were obtained from three to five independent imaging sets, each containing three to seven images per measurement condition. Area and length of the longest axis parameters were obtained from single-molecule analysis of three images per condition.

Before surface deposition and imaging, 18 ng of linearized DNA in 1× PBS buffer was incubated on ice with varying concentrations of DMSO (0, 2, 10, 20, 50 [% v/v]) in a total volume of 20 μL. Two independently prepared batches of linearized DNA were used for imaging and gave consistent results (Fig. S3). A comparison of incubation times (Fig. S4) found no significant effect and we chose an incubation time of 20 min for all conditions. After incubation in buffer with varying concentrations of DMSO, the samples were drop casted onto freshly cleaved poly-L-lysine (0.01% [w/v])-coated mica (Ted Pella, Redding, CA, USA) and incubated for 30 s, extensively rinsed with ultrapure water, and dried in a gentle stream of pure nitrogen gas (55,71,72).

AFM image processing and analysis

AFM images were processed with the MountainsSPIP 10 analysis software (Digital Surf, Besançon, France) applying line-by-line correction and polynomial background subtraction. End-to-end distances of individual DNA molecules were measured using the manual distance measurement tool of the software by drawing a line between the identified free ends of single DNA molecules (Fig. 5 *A*). The end-to-end distance is given by the horizontal distance between the first and the last point of the segment, along the defined path, measured on a horizontal plane. For end-to-end distance analysis we analyzed >125 individual DNA molecules for each measurement condition. Mean-squared end-to-end distances $<R^2>$ were directly computed from the data. In addition, we fit the distributions of end-to-end distances r obtained by AFM imaging using the probability function of a polymer chain in three dimensions (73,74):

$$Pr_{3D}\left(\langle R^2\rangle, r\right) = 4\pi r^2\left(\frac{3}{2\pi\langle R^2\rangle}\right)\exp\left(\frac{-3r^2}{2\pi\langle R^2\rangle}\right) \quad (1)$$

We note that the 3D Gaussian chain model is only an approximation to the situation in the AFM imaging. Therefore, Eq. 1 is primarily used as a convenient expression that fits the data (see also the next section).

In addition to the end-to-end distances, we obtained shape parameters for the area and the long-axis length of the DNA, again from the MountainsSPIP 10 software, using the manual shape analysis tool to draw contours around single DNA molecules manually. The area parameter is given by the projected horizontal area enclosed by the outline of the molecule. The long-axis length parameter is calculated by the software from the molecule's oriented bounding rectangle, which is parallel to the longest axis of the particle. With the area and long-axis length parameters, we can evaluate the compaction of every single DNA molecule that we could assign in the images and are not restricted to molecules with visible free ends, as is the case in the end-to-end distance analysis. The shape parameter analysis was applied to every single DNA molecule identified in a subset of three images per condition.

Models and scaling of the end-to-end distances of DNA

We use results from the statistical mechanics of semiflexible polymers to connect the DNA parameters determined from the MT force-extension measurements to the AFM results. The mean-squared end-to-end distance in 3D has been shown to be related to the bending persistence length L_P and contour lengths L_C via (75):

$$\langle R^2\rangle = 2\cdot L_P\cdot L_C\left(1 - \frac{L_P}{L_C}\left(1 - e^{-\frac{L_C}{L_P}}\right)\right) \quad (2)$$

Equation 2 describes the mean-squared end-to-end distance in 3D, while AFM images are recorded at a surface. To take into account the fact that AFM images are recorded in 2D, two limiting cases are often considered (76): 1) full 2D equilibration, where the chain completely equilibrates at the surface, and 2) and kinetic trapping, where the chain immediately "sticks" to the surface. Full 2D equilibration at the surface is expected to result in an equilibrated 2D chain for which L_P in Eq. 2 is replaced by $2\cdot L_P$. Therefore, a 2D equilibrated chain is predicted to have an approximately twofold larger value for $<R^2>$ than in 3D. Conversely, the kinetic trapping regime has been approximated by a simple, mathematical projection of the 3D chain onto 2D (75), in which case Eq. 2 is multiplied by 2/3. Importantly, both limits are approximations and real imaging conditions are likely between these extremes. It has been shown that our imaging conditions (deposition on PLL-mica) do not lead to 2D equilibration (76,77), but that the DNA conformations are, at least approximately, representative of the DNA structure in solution. Further, all three scenarios for $<R^2>$ (Eq. 2 for 3D, full 2D equilibration, and the 3D to 2D projection) predict the same scaling of the mean-squared end-to-end distance $<R^2> \sim L_P \cdot L_C$.

Coarse-grained Monte Carlo simulations of DNA

We performed Monte Carlo simulations of a modified worm-like chain (WLC) model, employing a simulation procedure similar to that used previously (59,78–80). The DNA is modeled as a chain of rigid bodies represented by right-handed reference frames (triads) akin to the rigid basepair model (80–83). The molecule is assumed to be inextensible, i.e., we consider a constant discretization length of $a = 0.34$ nm, corresponding to the length of 1 bp (84,85). Individual configurations are parametrized in terms of rotation vectors that transform adjacent triads into one another (for a detailed discussion see (80)). The three components of the vector, expressed in the local material frame, are identified with two local bending degrees of freedom (Ω_1 and Ω_2) and twist (Ω_3). We note that our simulations have freely rotating ends, unless otherwise noted, such that the twist does not influence the results.

Although this description allows for the consideration of sequence-dependent structure and elasticity (80–82), we ignore sequence effects and consider two types of basepair steps: duplex and bubble. Duplex steps are parametrized with a bending persistence length of $L_{P,duplex} = 43$ nm and a twist persistence length $C_{duplex} = 100$ nm, in agreement with values obtained from single-molecule experiments (59).

For bubble steps, which represent local defects or perturbations of the DNA helix induced by DMSO, we consider various bending stiffnesses $L_{P,bubble}$ with values ranging from 0.5 to 5 nm, which span the range of values reported for the persistence length of single-stranded DNA (86,87) and DNA loops (88). Similarly, bubble steps are modeled with a reduced twist stiffness of $C_{bubble} = 25$ nm. We note that values of C_{bubble} for the torsional stiffness in the range of 15–40 nm lead to similar agreement with experimental measurements. However, very low values of the twist stiffnesses, $C_{bubble} < 10$ nm, were found to be inconsistent with experiments as such values significantly alter the buckling behavior of supercoiled DNA even at very low bubble fraction and significantly extend the prebuckling regime.

Additionally, for duplex steps, we impose an intrinsic twist of $\omega_{0,duplex} = 34^\circ$, reflecting the natural helical winding of the molecule. For bubble steps, we introduce two modified values of intrinsic twist, $\omega_{0,bubble} = 17$ and 0°, corresponding to half and full unwinding of the affected segment, respectively. These elastic constants are incorporated into the elastic Hamiltonian of a N+1 basepair molecule as:

$$\beta E = \frac{1}{2a}\sum_{i=1}^{N}\left(L_{P,X}\left(\Omega_1^2 + \Omega_2^2\right) + C_X\left(\Omega_3 - \omega_{0,X}\right)^2\right)$$

where β is the inverse temperature, i.e., $\beta = 1/k_B T$, where k_B is Boltzmann's constant and T the absolute temperature, and X is either equal to bubble or duplex, depending on the type of the segment.

Simulations were conducted under varying conditions to explore the potential distribution and clustering of bubble segments. Bubble domains were modeled to vary in size, ranging from individual segments to full-phase separation, where all bubble segments are clustered into a single domain. Intermediate bubble sizes of 1, 3, 10, 25, 100, and 250 segments and full-phase separation were simulated to capture different degrees of bubble organization. For each bubble size, simulations are initiated with a randomly chosen but fixed distribution of bubbles. To account for variations in bubble positioning, we performed 20 independent simulations with different randomly chosen bubble locations for each set of simulation parameters. Since we do not know a priori how many bubbles there are and how their number depends on DMSO concentration (and possibly force and torque, etc.), we treat the fraction of bubbles f_{bubble} as an adjustable parameter. We simulated chains over a wide range of bubble fractions, $f_{bubble} = 0$–0.2, to identify which parameters are consistent with experimental observations. Ensembles of configurations are generated with a

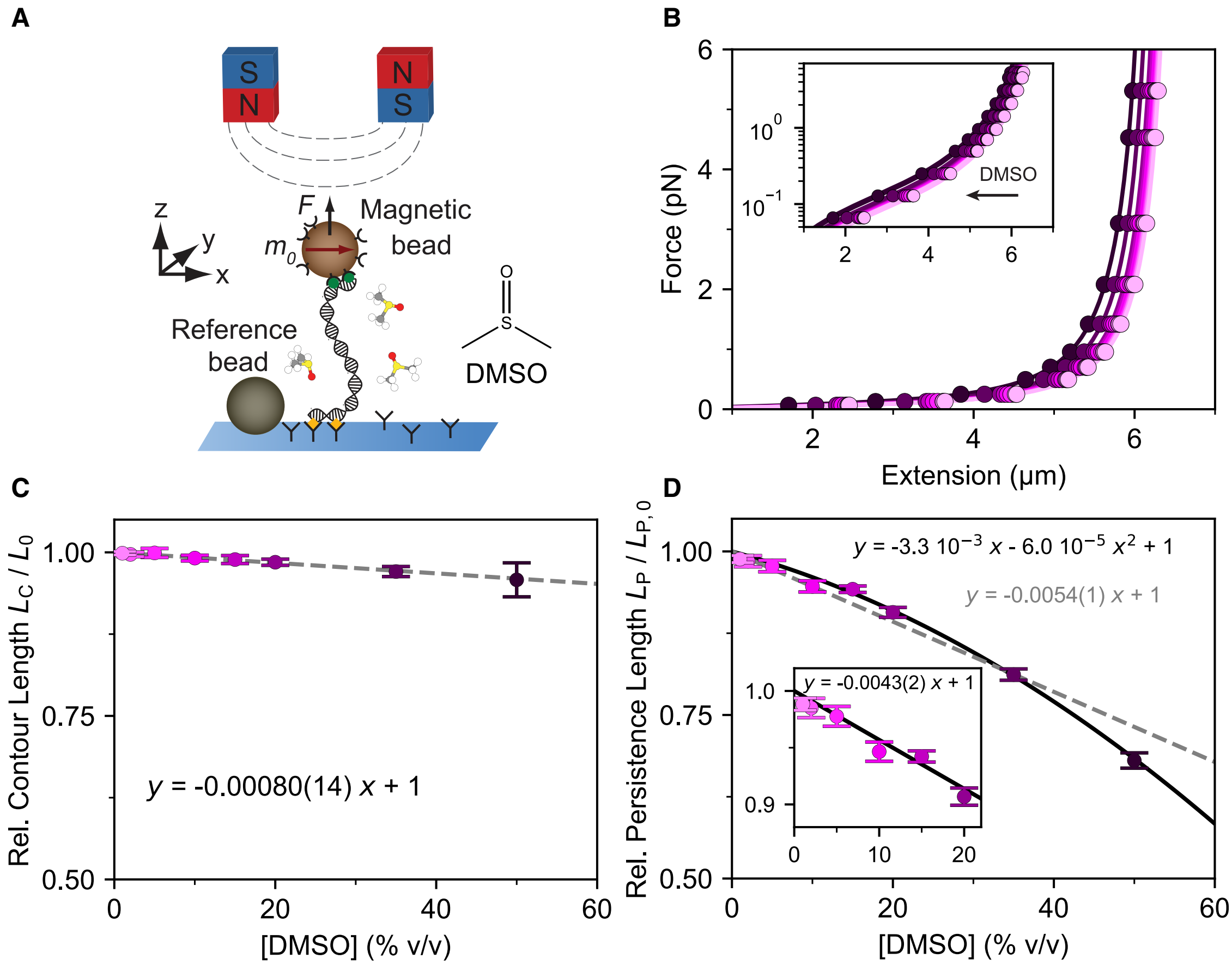

FIGURE 1 MT force-extension measurements reveal a linear decrease in contour and bending persistence length with increasing DMSO concentration. (*A*) Schematic of the MT measurement. DNA molecules are tethered between a surface and magnetic beads in a flow cell. Permanent magnets above the flow cell enable the application of stretching forces F and torques. (*B*) Force-extension measurements in the presence of increasing concentration of DMSO for 20.6 kbp DNA in PBS buffer. The DMSO concentrations are (from *light magneta* to *dark purple*) 0, 1, 2, 5, 10, 15, 20, 35, 50% (v/v). Solid lines are fits of the worm-like chain (WLC) model, treating the contour length L_C and bending persistence length L_P as fitting parameters. (*C*) Contour length L_C as a function of DMSO concentration was obtained from WLC fits as shown in (*B*). Data are normalized to the fitted contour length in the absence of DMSO (i.e., in PBS buffer), which is 6.9 ± 0.2 μm for our 20.6 kbp DNA construct. (*D*) Bending persistence length L_P as a function of DMSO concentration was obtained from WLC fits as shown in (*B*). Data are normalized to the fitted persistence length in the absence of DMSO (i.e., in PBS buffer), which is 43 ± 6 nm. The black line is a second-degree polynomial fit to the data. Inset shows a linear fit to the data up to 20% DMSO with a (0.43 ± 0.02%) decrease in L_P per vol %. Data in (*C*) and (*D*) are the mean ± SD and mean ± SE from at least 8 independent molecules, respectively. Fitted parameters are indicated as insets.

combination of cluster moves (crankshaft and pivot moves (59,79)). For the tweezer setup simulation, an additional force-dependent term $\beta\vec{f}\cdot\vec{R}$, where R is the end-to-end distance of the molecule, is introduced to the elastic energy (78). Each simulation was run for 10^8 Monte Carlo steps, which is much longer than the typical convergence sampling of 10^6–10^7 steps. To carry out the simulations we employed the previously developed Polymer Monte Carlo package PolyMC (available at https://github.com/eskoruppa/PolyMC) (59,79).

RESULTS AND DISCUSSION

To determine the effects of DMSO on the mechanical properties of DNA, in particular to test whether the presence of DMSO induces lengthening, unwinding, or a change in the bending stiffness of DNA, we performed single-molecule MT measurements (Fig. 1; materials and methods). In a first set of experiments, we measured the DNA extension at different levels of applied force for torsionally relaxed DNA. In a second set of measurements, we probed the response of DNA to systematic over- and underwinding at constant forces. We then probed DNA conformation in the presence of different DMSO concentrations in the absence of external forces by AFM imaging. Finally, we present coarse-grained Monte Carlo simulations to rationalize our findings from a microscopic view.

DMSO causes a gradual decrease of DNA bending persistence length

To investigate the effect of DMSO on DNA length and bending stiffness, we used MT to perform stretching experiments for increasing DMSO concentrations (0–60%) (Fig. 1 *A*). We first performed force-extension measurements on torsionally unconstrained DNA at stretching forces ≤5 pN: in this range, the response of DNA is

dominated by entropic stretching elasticity (89–91). The inextensible WLC model (89–91) describes the resulting force-extension curves well (Fig. 1 *B*) and for DNA in PBS without DMSO we find a contour length of $L_C = 6.9 \pm 0.2\ \mu m$ and a persistence length of $L_P = 43 \pm 6$ nm (mean $\pm$ SD from 86 molecules) in excellent agreement with previous measurements (51,91,92).

Next, we measured force-extension curves after addition of increasing amounts of DMSO (Fig. 1 *B*). From fits of the WLC model, we find a systematic decrease in the contour length (Fig. 1 *C*) and bending persistence length (Fig. 1 *D*) with increasing DMSO concentrations. The observed decrease in contour length is approximately linear and very small, <5% even at 50% DMSO. A linear fit gives a decrease in the apparent contour length of 0.08% per vol % DMSO, but we note that the change is essentially within experimental error.

In contrast, we find an overall still moderate, but systematic decrease in DNA bending stiffness for increasing DMSO concentrations (Fig. 1 *D*). For low and intermediate concentrations of DMSO, the decrease in bending persistence length with increasing DMSO concentration is linear, and from a linear fit up to 20% DMSO ($\chi^2_{red} = 1.3$) we find a (0.43 $\pm$ 0.02%) (the error is obtained from the covariance matrix of the fit) decrease in bending persistence length per vol % DMSO (Fig. 1 *D*, *inset*). For higher DMSO concentrations a larger dependence is observed (Fig. 1 *D*). The entire concentration range is less well described by a linear dependence, a linear fit up to 50% DMSO gives a (0.54 $\pm$ 0.01%) decrease per vol %-DMSO with a $\chi^2_{red} = 7$. We find that a second-degree polynomial provides a good description of the data ($\chi^2_{red} = 1.3$; Fig. 1 *D*).

Importantly, control measurements show that the change in bending persistence lengths is not due to the reduction in ionic strength due to dilution of the buffer upon addition of DMSO, since results within experimental error are obtained if the ionic strength and buffer concentration are kept constant upon addition of DMSO (Fig. S5). In addition, a decrease in ionic strength (upon dilution) would be expected to result in an increase and not decrease in bending persistence lengths (31,33,39–41). We note that, even when matching the ion and buffer concentration before and after addition of DMSO, as was done in our control measurements, addition of DMSO will still change the dielectric constant of the solution and thus our observation does not exclude a contribution of electrostatic interactions to the observed changes in DNA stiffness.

DNA stability and collapse in high DMSO concentrations

We tested the stability of DNA in moderate concentrations of DMSO by monitoring the force response of DNA for long periods of time (0.5–1.5 h) in varying concentrations of DMSO (see Fig. 2 *A* for 20% and Fig. S6 for 50% DMSO). The extension time traces show extension changes in response to changes in applied force, as expected from the WLC behavior discussed above, but exhibit no time dependence otherwise. In particular, no sudden decrease in extension or compaction of the DNA is observed in the range of forces investigated (0.07–5 pN). The extension at a given force remains constant and is reproducibly achieved in repeat measurements lasting >1 h (Fig. 2 *B*).

To investigate the behavior of DNA in the presence of very high concentrations of DMSO, we increased the concentration to 60% DMSO. Under these conditions, the DNA collapses against stretching force of 5 pN into a compact, condensed state (Fig. 2 *C*). This collapse is reversible: upon reintroduction of the initial buffer condition, the DNA returns to the extension measured before collapse in absence of DMSO (Fig. 2 *C*, *right panel*, *gray dashed line*). The observed collapse is likely related to a decrease in solvent quality of mixtures with high fractions of DMSO, as has been observed for lower DMSO concentrations in the presence of high concentrations of divalent metal ions (93) and in organic solvents and alcohols (45,46).

Since the emphasis in this work is on the properties of DNA under low and intermediate concentrations of DMSO, as are, e.g., typically used as additives in enzymatic assays, we limited our measurements to $\leq$50% DMSO in the remainder of this work. It might be an interesting future direction to investigate the mechanistic nature of this transition and to delineate its parameters in greater detail.

DMSO has only minor effects on DNA twist

Effects on the twist *Tw* of DNA can be monitored by control of the molecule's linking number *Lk*. In the case of torsionally constrained DNA, the linking number *Lk* can be directly controlled in the MT by rotation of the external magnets. We find the magnet orientation at which DNA is torsionally relaxed in PBS buffer in the absence of DMSO, which corresponds to the intrinsic helicity Lk_0 of dsDNA and which we refer to as zero turns, by systematically over- and underwinding the molecule (Fig. 3 *A*). At low applied stretching forces ($F = 0.25$ pN), an approximately symmetric response upon under- and overwinding is observed, with buckling transitions for negative and positive turns. Past the buckling transitions, a linear decrease in extension is observed upon further rotation of the magnets, corresponding to the formation of positive or negative plectonemic supercoils of the DNA strands (29,49). We quantify the change in the twist of DNA by extrapolating the slopes in the plectonemic regime (94), which provides the intersection points, which indicate the points where the molecule is torsionally relaxed. In contrast to the contour and persistence length, the twist of DNA is affected much less by increasing DMSO concentrations (Fig. 3 *B*). The intrinsic helicity in PBS remains unchanged, within experimental error, below 20% DMSO, which suggests that there are no large open

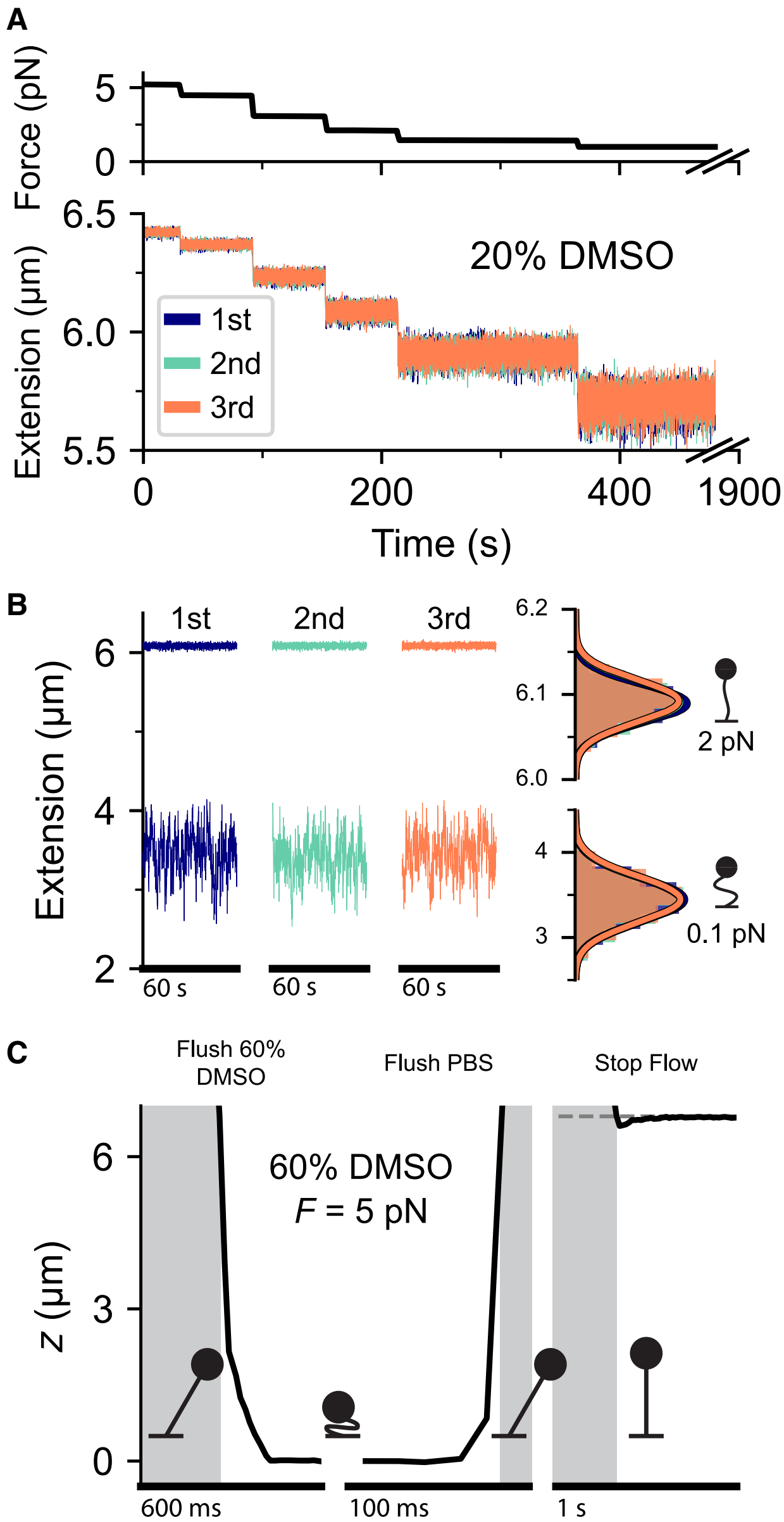

FIGURE 2 MT measurements reveal the effect of DMSO on the stability of DNA. (*A*) Force response of our 20.6 kbp DNA construct in 20% (v/v) DMSO. Top: forces applied to the molecule versus time. Bottom: extension of the molecule versus time. The data shown are from 3 subsequent force scans (indicated by the different colors), recorded over a period of time of 1.5 h. The response of the DNA remains highly consistent throughout the subsequent force cycles, such that traces are superimposable. (*B*) Sixty-second segments from extension time traces at the forces indicated on the right (0.1 or 2 pN) in 20% DMSO. The full extension time traces at 0.1 pN are significantly longer (300 s) than the segments shown. The behavior of the molecule remains unchanged, even after being at low force (0.1 pN) for a long period of time (5 min). Right inset shows the distribution of measured extensions for the total time trace at the given force. (*C*) Extension-time traces of DNA in the presence of 60% DMSO. DNA collapses against a constant 5 pN stretching force as a 60% DMSO solution is flushed into the MT flow cell. The DNA collapse is reversed upon flushing PBS buffer, when the molecule extends again to its initial length. The horizontal gray dashed line is the measured extension at 5 pN stretching force in PBS before DNA collapse. The scale bars indicate the duration of the segments. The vertical gray boxes indicate times when tracking of the magnetic bead is disrupted due to flushing for buffer exchange. Total flushing time of both 60% DMSO and PBS was ~8 min.

regions or extended melting bubbles. Above this concentration we observe a small decrease in the DNA twist, by (0.22 ± 0.08) degrees per basepair for 50% DMSO. We note that a reduction of DNA twist at high DMSO concentrations is both qualitatively consistent with changes of the double-helix due to reduced electrostatic screening and also with local melting of the DNA. DNA twist decreases with decreasing salt concentration (32,37); DMSO has a lower dielectric constant than water (≈47 for pure DMSO vs. ≈80 for water), which implies that DNA-DNA interactions would be increased with increasing DMSO concentrations. At the same time, local DNA melting is associated with unwinding of the helix (29,95,96). Both effects are predicted to cause DNA unwinding. We note that the observed decrease in twist disfavors a model whereby increased ion-DNA interactions overcompensate the increase in DNA-DNA repulsion upon addition of DMSO, since enhanced ionic screening would be expected to increase DNA twist.

From the rotation curves at low forces, $F = 0.25$ pN, we quantify the slopes of turns versus extension past the buckling points, i.e., in the negative and positive plectonemic regimes (Fig. 3 *C*). We find that at this low force, the slopes in the negative and positive plectonemic regime remain equal, within experimental error, for all DMSO concentrations except for 50% DMSO. In addition, the dependence on DMSO concentration can be accounted for by the change in bending persistence length (Fig. 1 *D*) using the scaling proportional to $L_P^{1/2}$ that is predicted by models of DNA supercoiling (50,59,97,98) (Fig. 3 *C*, *black line*).

DMSO facilitates torque-induced melting

Next, we measured the effect of DMSO on rotation-extension curves at different forces (0.25, 0.5, 0.8, 1 pN, Fig. 3), to monitor the increase in DNA melting by DMSO using MT. In the absence of DMSO, the rotation-extension curves of bare DNA are symmetric at forces below ≈1 pN, presenting buckling points for both negative and positive turns. At forces ≥1 pN, DNA buckles when overwound, but underwinding results in only a small decrease in extension, due to torque-induced melting, such that further unwinding is accommodated by DNA melting (29,70). At a force of ≈0.8 pN in PBS buffer, the DNA rotation curves are still essentially symmetric, but one notices increased fluctuations in the extension (Fig. 3 *E* and (59)).

In the presence of increasing concentrations of DMSO, the DNA rotation curves start to be asymmetric at lower forces compared with PBS buffer only, suggesting an increase in torque-induced melting in the presence of DMSO. This is consistent with the view that DMSO weakens the hydrogen

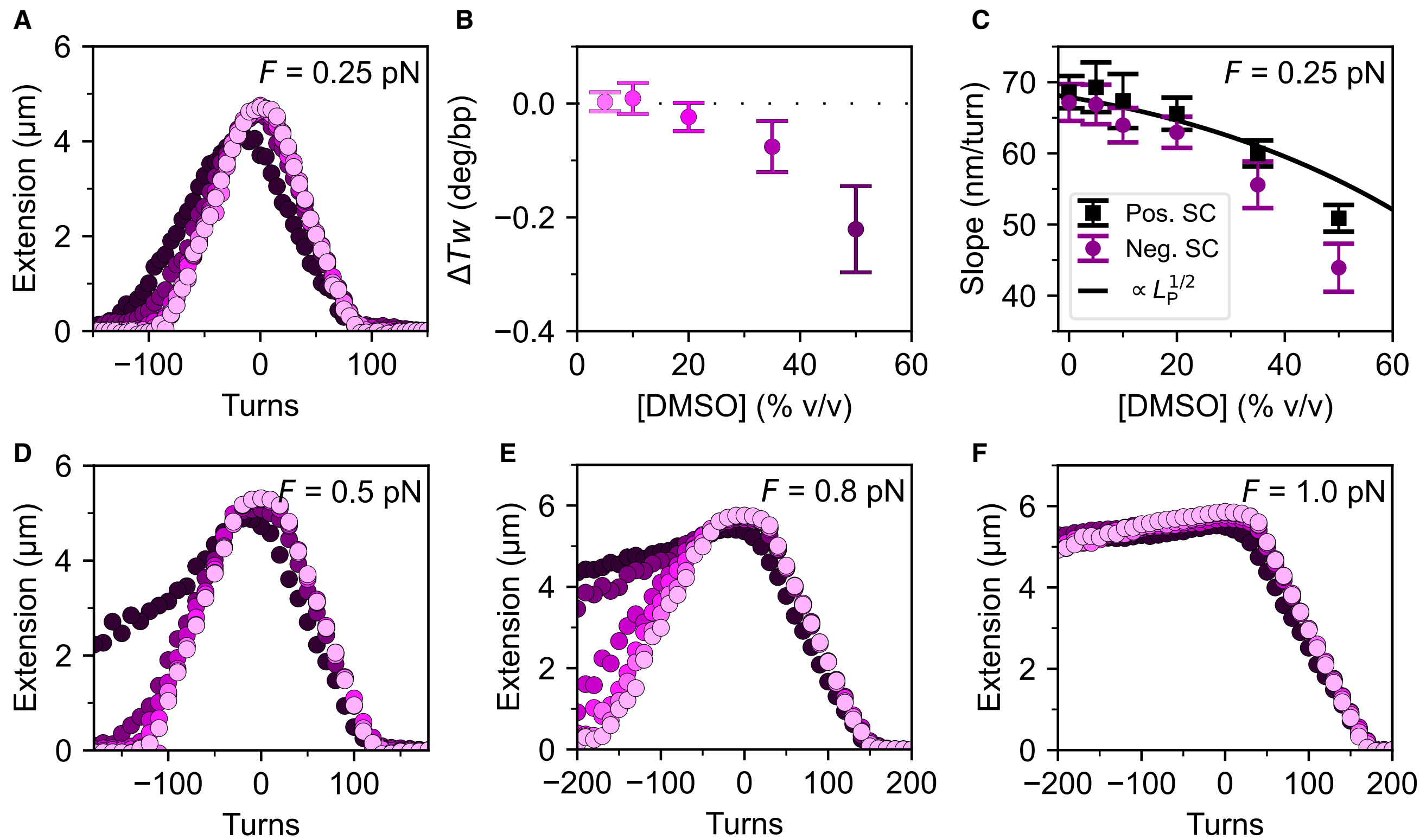

FIGURE 3 MT rotation extension measurements show that increasing DMSO concentrations facilitates melting but have only a small effect on DNA twist. (*A*) Rotation-extension measurements of a 20.6 kbp DNA in the presence of increasing concentration of DMSO (from *light magenta* to *dark purple*; same color code as Fig. 1). (*B*) Change in DNA twist as a function of DMSO concentration determined from the centers of rotation curves as in (*A*). The twist is measured relative to DNA in the absence of DMSO (i.e., in PBS buffer). (*C*) Slopes of the rotation-extension curves in the plectonemic regime at $F = 0.25$ pN for positive and negative turns (relative to relaxed DNA). Data in (*B*) and (*C*) are the mean ± SD from at least 5 independent molecules (at least 11 for DMSO concentrations ≤10%). The solid line is the square root of the quadratic fit to the $L_P/L_{P,0}$ data from Fig. 1 *E*, normalized to the slope in the absence of DMSO (i.e., in PBS buffer). A dependence of the slope in the plectonemic regime on $L_P^{1/2}$ is predicted by models of DNA supercoiling (50,59,97,98). (*D–F*) Rotation-extension curves for 20.6 kbp DNA as a function of DMSO concentration at forces of 0.5 pN (*D*), 0.8 pN (*E*), and 1.0 pN (*F*). While curves at low forces, e.g. (*A*) are approximately symmetric around zero turns, the rotation curves at higher forces and higher DMSO concentration are asymmetric, exhibiting lower (absolute) slopes for negative turns than at positive turns, due to torque-induced melting. It is apparent that the onset of torque-induced melting occurs at lower forces for higher DMSO concentrations.

bonds between the bases, which would correlate to destabilization of the dsDNA form observed in the MT, in line with the reported decrease in the melting temperature of DNA in the presence of DMSO (23,99).

AFM imaging reveals a gradual compaction of DNA upon addition of DMSO

MT measurements constrain the ends of the DNA and stretch the molecule, which might potentially suppress the effects of compaction, local melting, or kinks on its conformations. Therefore, we performed AFM imaging experiments to visualize the conformations of linear dsDNA in the absence of external forces and torques after surface deposition (76,100,101) in near-physiological PBS buffer with varying DMSO concentrations ranging from 0 to 50% (materials and methods). Our AFM images, recorded in tapping mode in air, clearly visualize individual DNA molecules, showing the typical "worm-like chain" conformations of semiflexible polymers and suggest a small, but systematic compaction of the DNA conformations with increasing DMSO concentration (Fig. 4).

To quantify the shape and compaction of individual DNA molecules, we quantified three parameters using the MountainsSPIP 10 analysis software (materials and methods): 1) We obtained end-to-end distances using the manual distance analysis tool by drawing straight lines between the free ends of single DNA molecules (Fig. 5 *A*). The end-to-end distance is a commonly used parameter to analyze the size of linear polymers in solution. End-to-end distances give an indication of the structural behavior of DNA and with clear predictions from polymer physics and can be linked to parameters from MT experiments via $\langle R^2 \rangle \sim L_P \cdot L_C$ (Eq. 2, materials and methods). 2 and 3) To evaluate and confirm the compaction of DNA without the necessity to identify the ends, which can only be analyzed for ~40–50% of the molecules (Fig. S7), we additionally obtained area and long-axis parameters from the AFM images. Using the manual shape analysis tool of the MountainsSPIP10 software, we drew contours around

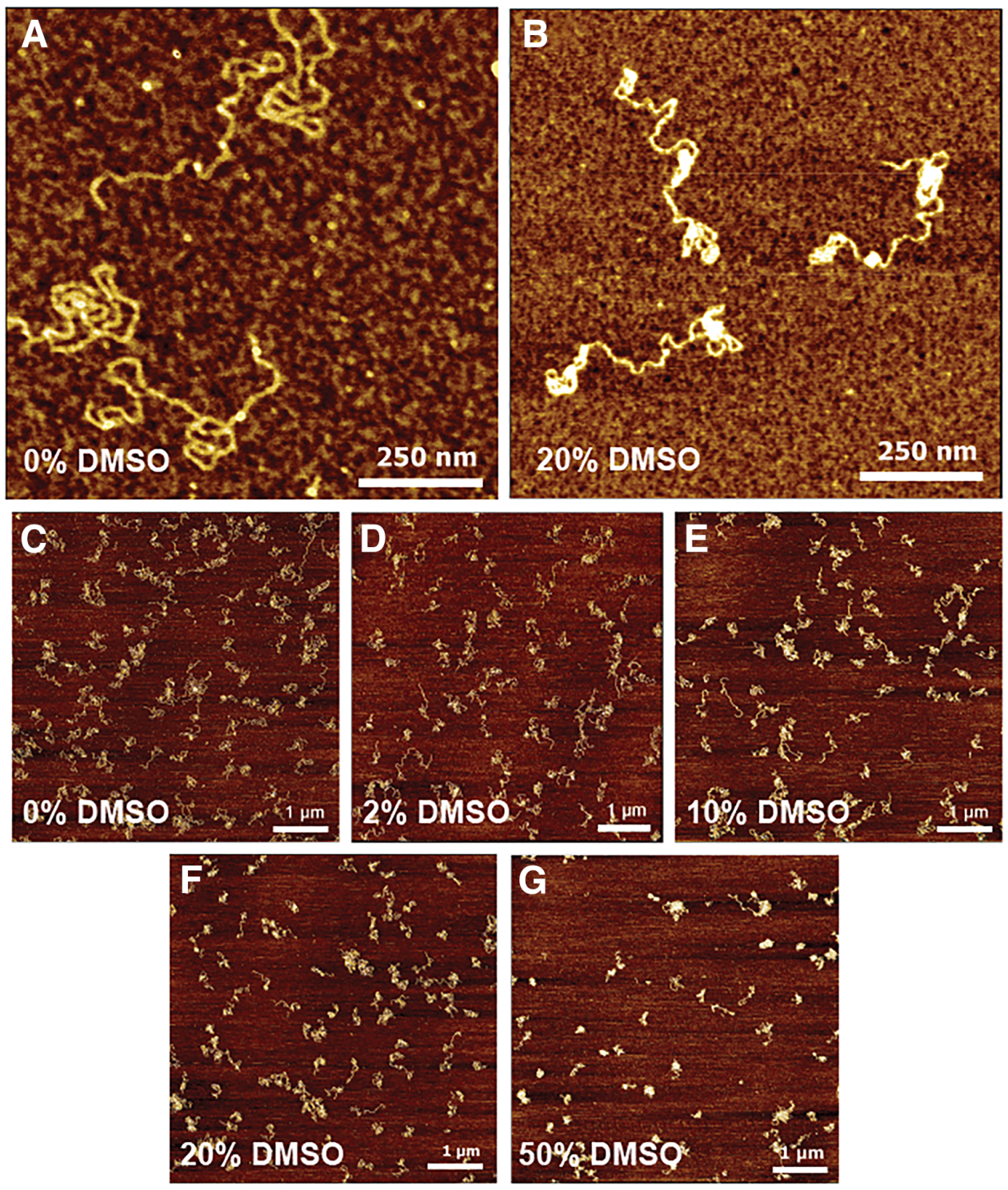

FIGURE 4 AFM imaging probes DNA in the presence of DMSO. Topographic images of linearized pBR322 DNA on PLL-mica measured in air without DMSO (*A* and *C*) and with DMSO concentrations ranging from 2 to 50% (*B* and *D–G*). It is visible that DNA moderately compacts in high DMSO conditions (≥20%) while differences in DNA structure and compaction are not distinguishable by eye for DMSO concentrations below 20%. High-resolution images of DNA are shown for PBS only (*A*) and for 20% DMSO (*B*).

individual DNA molecules (Fig. 5 *A*) and obtain the output parameters from the software. While less readily linked to polymer theory, the area and long-axis length parameters provide a control to quantitate systematic changes of DNA conformations upon increasing DMSO concentration.

All three parameters show similar trends, namely a moderate but gradual decrease in size upon increasing DMSO concentration (Fig. 5). The area of the molecules shrinks to 58% of its area in the 50% DMSO condition in comparison with no DMSO treatment and decreases approximately linear with 0.71% per %-DMSO over the whole range. The mean long-axis length decreases to 70% of its length at 50% DMSO with 0.51% per %-DMSO, indicating moderate systematic compaction of the DNA molecules (Fig. 5 *B*). The effect of DMSO on the end-to-end distances $<R^2>$ is described in detail in the next section.

DMSO induces a gradual decrease of the end-to-end distances

To quantify the DNA conformations visualized by AFM imaging, we identify the ends of the DNA molecules and measure the corresponding end-to-end distances (materials and methods; Fig. 5 *A*). While the end-to-end distances could not be traced for all molecules visible in the AFM images (~40–50%, Fig. S7), in particular due to obscured or overlapping ends, we obtained a similar number of traced molecules for each DMSO condition (≥125 molecules per condition). The resulting end-to-end distance distributions show the typical positively skewed distributions previously observed (76) for DNA by AFM imaging and are well described by the Gaussian chain model shown in Eq. 1 (Figs. 5 *C* and S8). The distributions shift to smaller end-to-end distances with increasing DMSO concentrations and we find that the distributions for all investigated DMSO concentrations are significantly different from the PBS-only conditions, as assessed both using two-sample Kolmogorov-Smirnov tests to compare the full distributions and using two-sample two-tailed *t*-tests to compare the means (Table S1). Overall, we observe a moderate decrease in mean-squared end-to-end distance $<R^2>$ with increasing DMSO concentrations (Fig. 5 *D*), which is reasonably well described by a linear fit. Fitting a straight line, we obtain a slope corresponding to a change by 1.2% decrease in $<R^2>$ per %-DMSO. Using the $<R^2>$ values obtained from fits of the Gaussian chain model (Eq. 1) instead of the directly computed $<R^2>$ values gives similar results (Fig. S9).

Comparison of MT and AFM results for DNA conformations with increasing DMSO

We can use results from the theory of semiflexible polymers to connect the results of AFM and MT force spectroscopy (materials and methods). A complication in the interpretation of AFM images is that they are recorded for molecules deposited on a surface and that surface deposition conditions can influence what conformations are observed. It has been shown that our imaging conditions (deposition on PLL-mica) do not lead to 2D equilibration (76,77). For simplicity, we use the 3D prediction (materials and methods, Eq. 2) for the DNA scaling and find reasonable agreement with the experimentally determined values: In PBS only, we measured experimentally $<R^2>^{1/2} = 393 \pm 9$ nm (mean ± SE), while the 3D model predicts $<R^2>^{1/2} = 352 \pm 24$ nm, where we assume a contour length of 0.34 nm/bp and use L_P and its propagated error from the MT experiments.

Combining the dependences of L_P and L_C on DMSO from the MT data, we predict a reduction in $<R^2>$ by 0.62% per %-DMSO, which is smaller but of similar magnitude compared with the experimentally determined slope of 1.2% decrease of $<R^2>$ per %-DMSO (Fig. 5 *D*) and the change in $<R^2>$ values determined from the Gaussian fit (Eq. 1) that decreases by 0.99% per %-DMSO (Fig. S9). Similarly, using the L_P and L_C determined from MT measurements directly to predict $<R^2>$ at different DMSO concentrations via Eq. 2 finds values close to the data

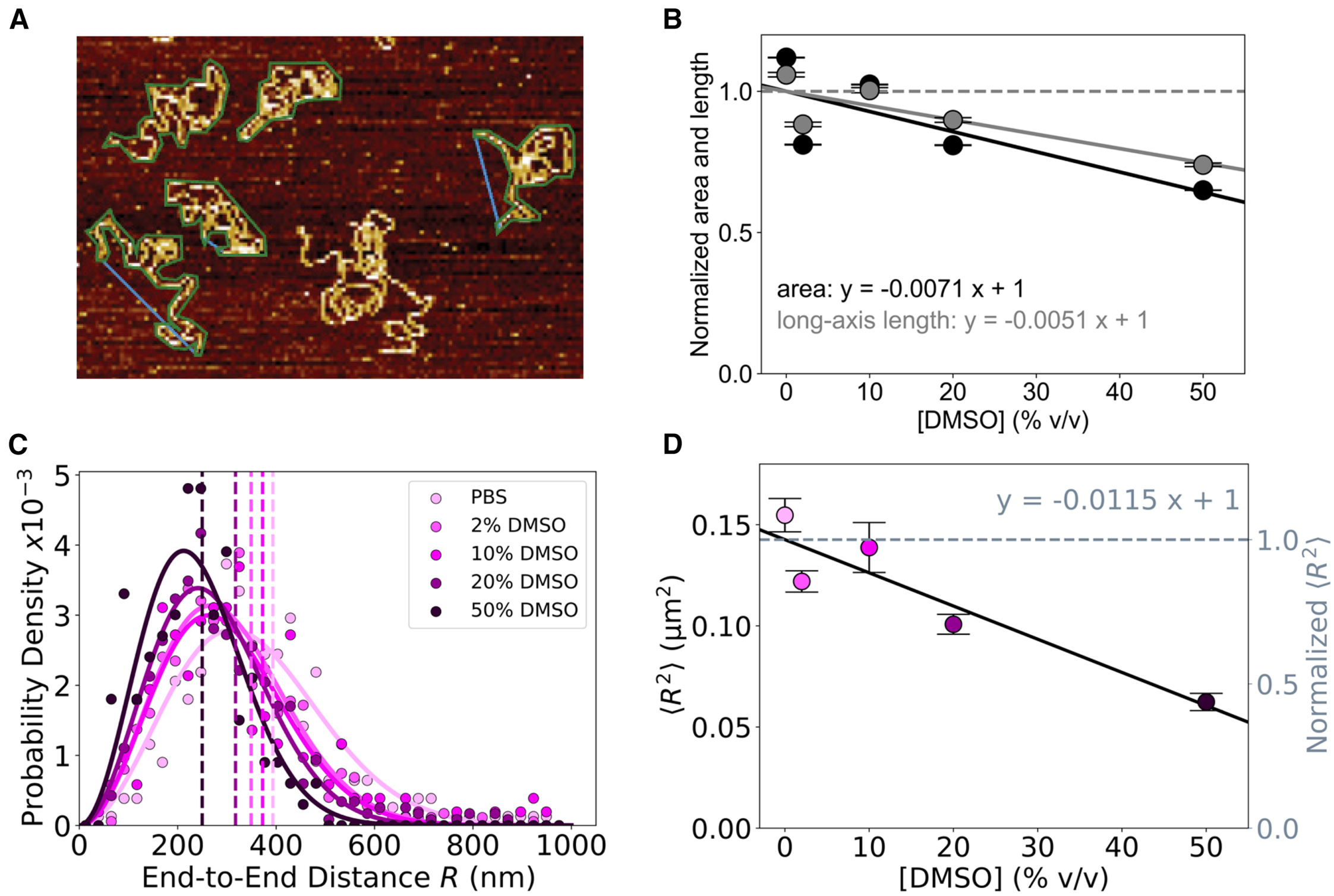

FIGURE 5 Quantification of AFM images reveals a moderate compaction of DNA in the presence of DMSO. (*A*) Topographic AFM image of linearized pBR322 DNA (4361 bp) in PBS on PLL-mica measured in air. End-to-end distances were measured manually using the manual measurement distance tool of the MountainsSPIP 10 software and are indicated by blue lines in the image. Green contours around DNA molecules show how we used the manual shape analysis tool of the MountainsSPIP 10 software to obtain shape parameters such as area and long-axis length. (*B*) Long-axis length (shown in *gray*) and area (shown in *black*) parameters as a function of increasing DMSO. The data are normalized to the 0% DMSO value from the fit. The solid lines indicate a linear fit with a negative slope corresponding to a decrease in area of 0.71% (*black line*) and long-axis length of 0.51% (*gray line*) per %-DMSO. (*C*) Histograms of DNA end-to-end distances with increasing concentrations of DMSO (from 0%, *magneta*, to 50%, *dark purple*) and fits of the end-to-end distribution of a Gaussian chain (*solid lines* in the corresponding colors). RMS values are indicated as colored dashed lines and show a trend of decreasing end-to-end distance of the DNA upon increasing DMSO concentration. (*D*) Mean-squared end-to-end distances $<R^2>$ as a function of increasing DMSO concentration. The right axis shows values relative to the 0% DMSO value from the fit. The black line indicates the linear fit with a negative slope corresponding to ∼1.2% decrease of mean-squared end-to-end distance per %-DMSO. Symbols and error bars in (*B*) and (*D*) are the mean ± SE.

determined from AFM imaging (Fig. S10). In summary, we find an overall consistent view of the changes in DNA mechanics upon addition of DMSO from MT force spectroscopy and AFM imaging analysis, which probes DNA in the absence of applied forces or torsional constraints. Nonetheless, the effects on DNA seen by AFM imaging are moderately larger than what is observed by MT force spectroscopy, which might indicate that applied forces reduce the impact of DMSO on DNA mechanics. For example, the applied force might suppress the effects of local interactions on overall stiffness or reduce electrostatic effects by suppressing higher curvature conformations.

Coarse-grained Monte Carlo of DNA with flexible segments

To investigate the observed effects of DMSO from a mechanistic and more microscopic perspective, we turned to coarse-grained Monte Carlo simulations and introduce a minimal model that features local defects, which we refer to as bubbles. We model DNA as a discrete WLC with two types of inextensible segments each of size 0.34 nm, corresponding to the length of one basepair (materials and methods; Fig. 6 *A*). One type of segment represents dsDNA with a persistence length of 43 nm (matching the force-extension measurements of DNA in PBS buffer) and the second type of segment represents DMSO-induced defects, which are WLC segments of strongly enhanced flexibility. For simplicity we omit enthalpic stretching and, thus, assume the discretization length to be constant. The bubbles represent regions of local basepair disruption and have significantly lower bending stiffness $L_{P,bubble}$ (with $L_{P,bubble} << L_P$), but no intrinsic curvature (which has been considered previously in models of kinked DNA (54)). While the fraction f_{bubble} and the location of the bubbles remains fixed throughout each individual simulation, the effect of positional bubble fluctuations is mimicked by repeating simulations for each set of parameters for many different bubble distributions. Furthermore, our and previous (22) experimental data suggest that even in 50% DMSO DNA remains predominantly double stranded, suggesting

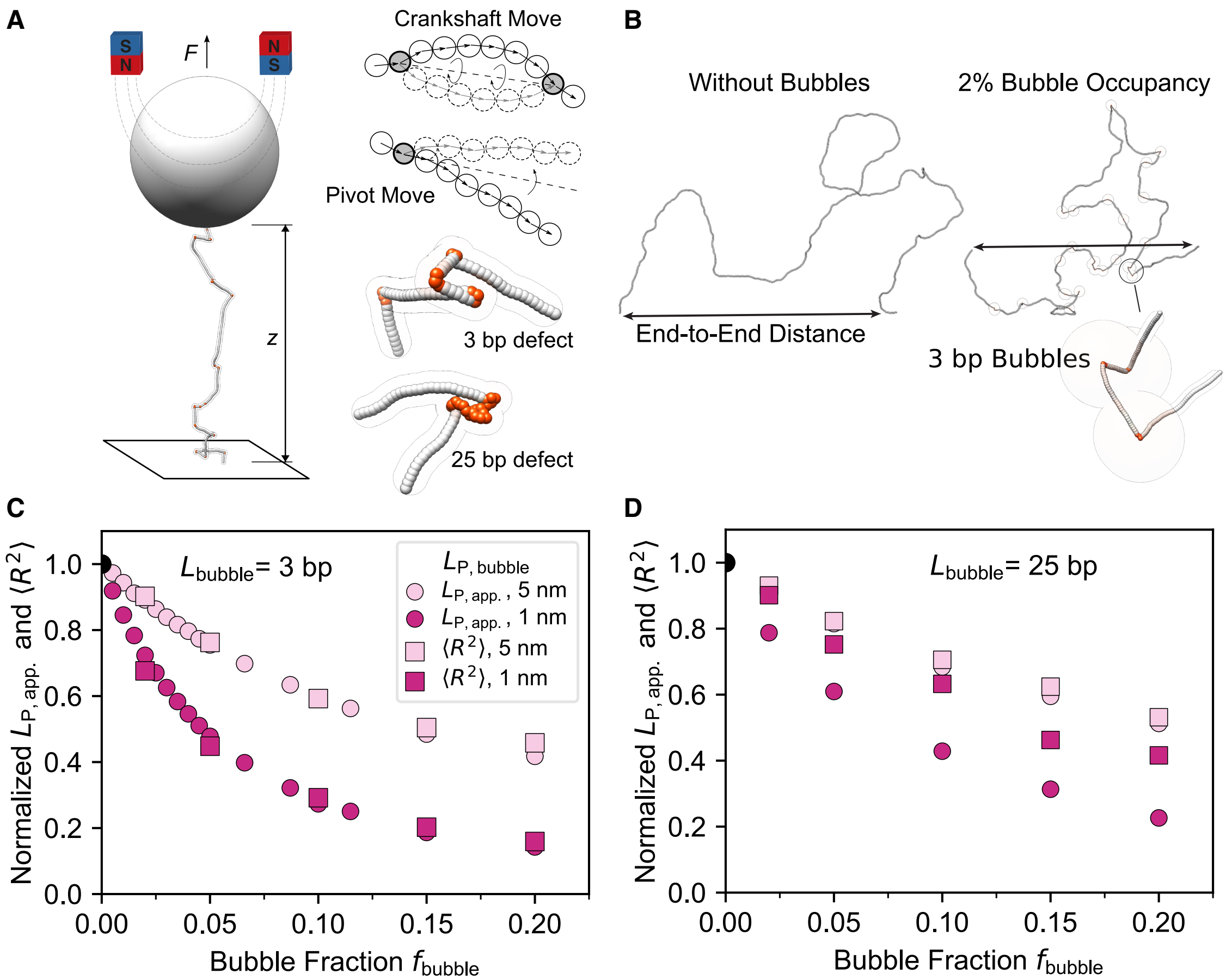

FIGURE 6 Coarse-grained simulations of kinkable DNA under external forces and in free solution. (*A*) Schematic of the coarse-grained MC simulations. The DNA is represented as a chain of beads connected by springs that set the bending stiffness of the chain. In force-extension simulations the ends of the chain are constrained and a constant force in *z*-direction is applied, mimicking MT measurements. Crankshaft and pivot moves ensure efficient sampling in the MC protocol (see Materials and Methods). Examples of flexible segments or bubbles are highlighted in red. (*B*) Snapshots of coarse-grained MC simulations of 4 kbp DNA in free solution. End-to-end distances are annotated in the plots. (*C* and *D*) Normalized bending persistence length obtained from fits of the WLC model to simulation data of 8 kbp DNA and normalized mean-square end-to-end distances $<R^2>$ from simulations of 4 kbp DNA in free solution. The apparent bending stiffness decreases systematically with increasing fraction of bubbles and with decreasing persistence length of the flexible segments. $<R^2>$ decreases systematically with increasing fraction of bubbles and with decreasing persistence length of the flexible segments. For bubbles of 3 bp the DNA remains well described by the WLC and obeys the dependence of $<R^2> \sim L_P \cdot L_C$. For larger bubbles, i.e., 25 bp, the DNA is less well described by the WLC for a small persistence length of the flexible segments and shows a decreased dependence of bubble fraction. Similar simulations for other parameters are shown in Figs. S11–S15.

that f_{bubble} is small, i.e., $f_{bubble} \ll 1$. Likewise, rather than assuming a certain bending stiffness for the bubble segments, we repeat simulations for values of $L_{P,bubble}$ ranging from 0.5 to 5 nm.

Coarse-grained Monte Carlo simulations quantify the effect of DNA bubbles on force-extension measurements and on DNA in free solution

To enable comparison with our MT measurements and AFM imaging data, we simulated the force-extension response of DNA and its conformation in free solution, using the coarse-grained model introduced in the previous sections (Figs. 6, *A*, *B* and S11). The simulated force-extension response of fully dsDNA is in excellent agreement with the WLC model and recovers the input value of L_P from a fit to the simulated data for bare DNA (Figs. S11 and S12).

We then introduce increasing numbers of flexible segments or bubbles, i.e., we simulated different f_{bubble} for a range of values of stiffnesses $L_{P,bubble}$ and sizes L_{bubble} of the flexible regions. We find that, for small bubbles ($L_{bubble} \leq 10$ bp), the simulated force-extension data remain well described by the force-extension expression of the WLC model (Figs. 6 *C*, S11, S12, and S13). In contrast, large bubble sizes (L_{bubble} > 25–100; Figs. 6 *D* and S11) lead to deviations from the WLC model, at odds with the experimental findings (Fig. 1). Fits of the WLC to the simulated data find reduced (effective) bending persistence lengths with 1) increasing bubble fraction, 2) decreasing bubble stiffness $L_{P,bubble}$, and 3) decreasing bubble size

L_{bubble} (Figs. 6, *C*, *D* and S12). We note that, since for large bubbles the simulated DNA does not behave like a WLC, the fitted apparent L_P is difficult to interpret.

Introducing bubbles into the simulated chains in the absence of applied forces, similarly leads to a reduction of the measured 3D end-to-end distance $<R^2>$ (Figs. 6, *C*, *D* and S14). Again, the end-to-end distances decreases with 1) increasing bubble fraction, 2) decreasing bubble stiffness $L_{P,bubble}$, and 3) decreasing bubble size L_{bubble} (Figs. 6 *C* and S15). We find that, for large bubble sizes (L_{bubble} > 25–100), $<R^2>$ decreases both in absolute value (Fig. S15) and relative to the zero DMSO conditions (Fig. 6 *D*) too little to account for the experimental observations. For small bubble sizes, we observe a reduction in $<R^2>$ broadly consistent with experiments and of similar relative magnitude as the reduction in the apparent bending persistence length (Fig. 6 *C*). Therefore, both the results from force-extension simulations and from simulations of molecules in free solution suggest that only models with small and dispersed bubbles are consistent with the experimental data. This conclusion is further reinforced by rotation-extension simulations, i.e., control simulations with constrained linking number, which qualitatively align with tweezer measurements for small bubbles but show significant deviations for large bubbles (Fig. S16).

Furthermore, we find that simulations with bubble stiffnesses >2 nm would require bubble fractions >10% (f_{bubble} > 0.1) to match the experimentally observed reductions in apparent bending stiffness and $<R^2>$, which is likely unrealistic. In addition, we find that the dependencies of the apparent bending persistence lengths and $<R^2>$ are convex with increasing bubble fraction (Figs. 6, *C*, *D* and S15), in particular at large bubble fractions. Conversely, the reduction of the experimentally determined values of the bending persistence lengths and $<R^2>$ are approximately linear (or even slightly concave, Fig. 1 *D*). Together, this suggests again that likely small and flexible bubbles with a small bubble fraction are most consistent with the experimental data and/or that the dependence between DMSO concentration and bubble fraction is superlinear.

Taken together, our coarse-grained simulations show that introducing flexible segments into the WLC model can account at least semiquantitatively for the experimentally observed reduction in apparent L_P and $<R^2>$. The simulations find that small and disperse bubbles with a stiffness similar to $L_{P,bubble}$ ~1 nm give the best agreement with experimental data. For these parameters a small fraction of bubbles (~3–4% at 50% DMSO) is sufficient to account for the experimental observations up to 50% DMSO. A small fraction of bubbles, in turn, is consistent with the fact that we do not observe substantial unwinding of the helix from rotation-extension curves and bubbles are not observed by AFM imaging.

CONCLUSION

We have used highly complementary single-molecule methods to probe the effect of DMSO on DNA conformations and mechanics. MT force spectroscopy, where the ends of the DNA are constrained and external forces applied, and AFM imaging, where DNA is visualized after equilibration in free solution, provide an overall consistent view. The bending persistence length, mean-squared end-to-end distances, and shape parameters such as areas and long-axis lengths all decrease systematically with increasing DMSO concentration (Fig. 7). While very high DMSO concentrations (>50% DMSO in near-physiological buffer) lead to a collapse of DNA, the observed changes are gradual and approximately linear up to 50% (Fig. 7). For low DMSO concentrations, <10%, the observed changes are minor, of the same order as typical measurement errors in single-molecule measurements. Our observations validate the—often implicit—assumption to neglect the effect of DMSO on DNA in single-molecule assays, where DMSO is introduced in concentrations usually below 10%, e.g., upon adding fluorescent dyes from a DMSO-containing stock (8,102) and are also consistent with the observation that higher-order DNA origami structures remain stable in up to 30–40% DMSO (26).

Our measurements are at odds with a recent report that finds a stronger reduction of end-to-end distances and bending persistence lengths upon addition of DMSO (27). We note that the measurements of Xu et al. for linear DNA are performed at very low ionic strength (1 mM Tris-HCl buffer only), which likely partially destabilizes the DNA and makes it particularly susceptible to the effects of DMSO, as has recently been observed for DNA origami structures (26). Consistent with this view, Xu et al. observe significant melting of the dsDNA already at concentration of 10% DMSO and complete denaturation at 50% DMSO (27). In contrast, our measurements are all performed under matching conditions in physiologically relevant buffer (~150 mM monovalent salt) and the DNA remains predominantly double-stranded for all DMSO concentrations tested.

Our coarse-grained Monte Carlo simulations rationalize the observed effects, at least qualitatively, in the light of a simple physical model: Introducing softer, much more bendable segments termed bubbles into the stiffer chain representing dsDNA leads to a reduction of the apparent bending persistent length and mean-squared end-to-end distance. Comparison of the model with our experimental observations suggests that the flexible bubbles are dispersed throughout the chain and remain relatively small. Under those conditions, only a small fraction of bubbles is sufficient to account for the experimentally observed reductions in apparent L_P and $<R^2>$.

It will be interesting to see whether future higher-level (all-atom MD or even quantum) simulations can provide a more detailed, atomistic view of how DMSO locally alters

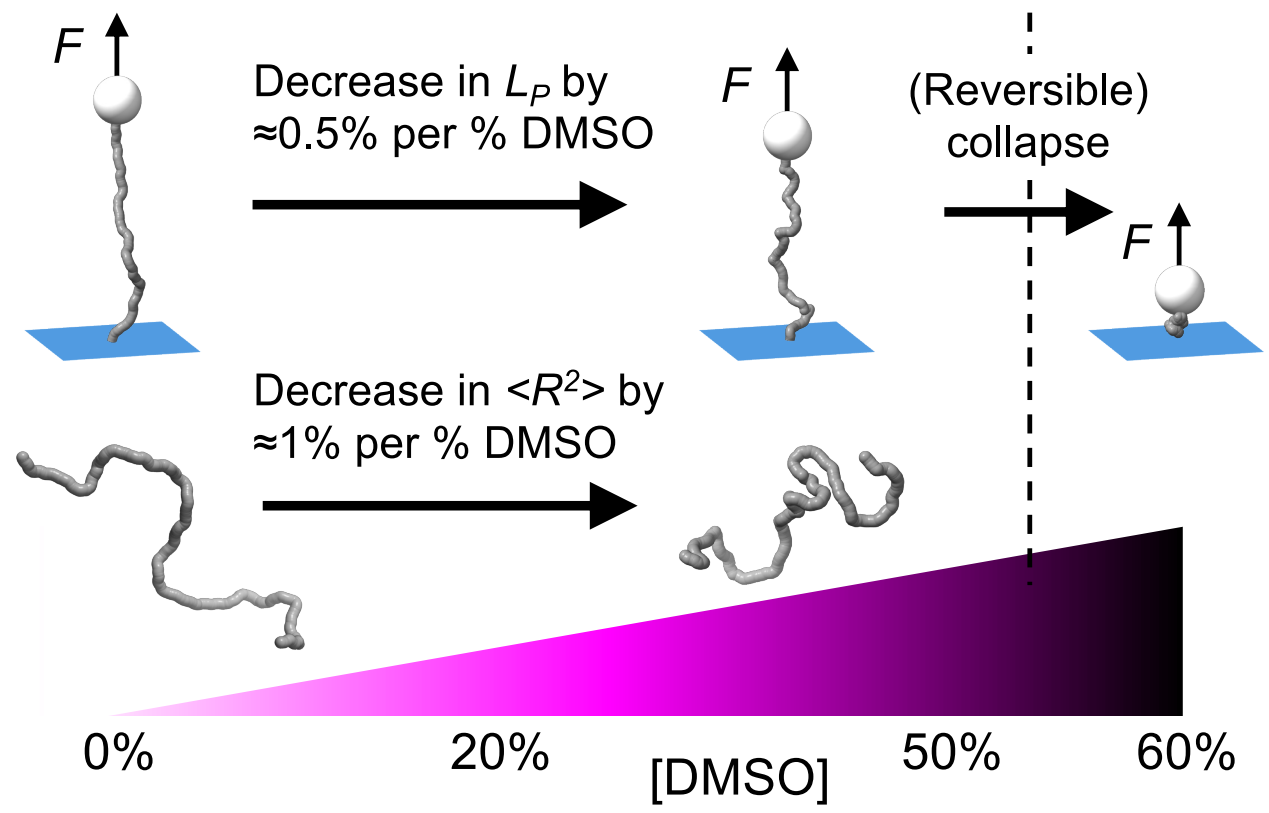

FIGURE 7 Summary of the effects of DMSO on DNA. Schematic summary of the effects of DMSO on DNA observed by MT force spectroscopy (*top*) and AFM imaging (*bottom*) in this work. Up to 50% DMSO, changes are gradual and approximately linear with DMSO leading to a reduction in (apparent) bending stiffness L_P and mean-squared end-to-end distance <R^2>. Above 50% DMSO, we observe a reversible collapse of DNA even against external forces.

DNA structure. Higher-level simulations could also address to what extent changes in the electrostatic environment contribute to the observed effects on DNA. While DMSO is still relatively polar and its dielectric constant ($\varepsilon \approx 47$) is larger than that of many other organic solvents, its addition to aqueous buffer decreases the dielectric constant, which in turn is expected to increase electrostatic interactions and alter the complex interplay of DNA-DNA, DNA-ion, ion-ion, and solvent interactions. For ethanol, a recent study suggests that increased DNA-ion interactions overcompensate increases in DNA-DNA repulsion and explain the reduction in bending stiffness by ethanol (48). However, while ethanol ($\varepsilon \approx 24$) has a lower dielectric constant than DMSO, its effect on bending stiffness is approximately twofold less than that of DMSO (using very similar salt concentrations than was used in this study). In addition, the observed decrease in DNA twist at high DMSO concentrations also suggests that an increase in ionic screening is unlikely to be the dominant effect for DMSO, as DNA twist would be expected to increase and not decrease with increased ionic screening (32,37).

We anticipate that the trends observed in our model will also be useful to interpret other types of experimental situations where DNA is locally rendered more flexible, e.g., by increased temperature or DNA damage. Our findings enable us to provide practical advice for common lab uses of DMSO. In addition to amplification and toposisomerization assays, which already frequently use DMSO at low concentrations, the observed moderate reduction of DNA stiffness might make it a beneficial additive in applications were an enhancement of DNA flexibility is expected to be desirable, e.g., in DNA packaging and delivery. Conversely, the fact that the changes to DNA mechanics are small (in the range of a few percent) if low enough concentrations (<10–20% DMSO) are used (Fig. 7), provides quantitative justification for neglecting changes to the DNA and its supercoiling, looping, and dynamic behavior after DMSO is added as a component of the buffer, e.g., after adding dyes or other small molecules that use DMSO as a solvent (7,8,102–106). In summary, our results provide quantitative parameters and a baseline understanding of how DMSO affects DNA conformations that will aid the interpretation and modeling of more complex biophysical and biochemical assays involving DMSO.

ACKNOWLEDGMENTS

We thank Dave van den Heuvel, Elleke van Harten, and Roy Hoitink for laboratory support and Gerhard Blab, Alptuğ Ulugöl, Willem Gispen, Nadine Schwierz, and Tor Sewring for useful discussions. This work was supported by Utrecht University, by the European Research Council Consolidator Grant "ProForce," a Feodor-Lynen fellowship from the Alexander von Humboldt Foundation, and the Deutsche Forschungsgemeinschaft (DFG, German Research Foundation) under Germany's Excellence Strategy - EXC-2068 – 390729961.

AUTHOR CONTRIBUTIONS

Conceptualization, K.R.S., C.K., E.S., and J.L.; data analysis, K.R.S., C.K., E.S., S.D.P., P.J.K., W.V., and H.S.; investigation (MT measurements), K.R. S.; visualization, K.R.S. and C.K.; writing – original draft, K.R.S., C.K., E. S., C.K., and J.L.: writing – review & editing, E.S., S.D.P., P.J.K., W.V., and H.S.; investigation (AFM measurements), E.S.; investigation (MC simulations); supervision, E.S., S.D.P., P.J.K., W.V., H.S., and J.L.; funding acquisition, J.L.; project administration, J.L.

DECLARATION OF INTERESTS

The authors declare no competing interests.

SUPPORTING MATERIAL

Supporting material can be found online at https://doi.org/10.1016/j.bpj.2025.06.041.

REFERENCES

1. Yu, Z.-W., and P. J. Quinn. 1994. Dimethyl sulphoxide: A review of its applications in cell biology. *Biosci. Rep.* 14:259–281.
2. Rawls, W. F., L. Cox, and E. S. Rovner. 2017. Dimethyl sulfoxide (DMSO) as intravesical therapy for interstitial cystitis/bladder pain syndrome: A review. *Neurourol. Urodyn.* 36:1677–1684.
3. Tomoe, H. 2023. Dimethyl sulfoxide : A review of pharmacology and clinical effect on interstitial cystitis/bladder pain syndrome. *Continence.* 8:101058.
4. Rössberger, J., M. Fall, and R. Peeker. 2005. Critical appraisal of dimethyl sulfoxide treatment for interstitial cystitis discomfort, side-effects and treatment outcome. *Scand. J. Urol. Nephrol.* 39:73–77.
5. Jacob, S. W., and R. Herschler. 1986. Pharmacology of DMSO. *Cryobiology.* 23:14–27.
6. Santos, N. C., J. Figueira-Coelho, …, C. Saldanha. 2003. Multidisciplinary utilization of dimethyl sulfoxide: pharmacological, cellular, and molecular aspects. *Biochem. Pharmacol.* 65:1035–1041.

7. Ganji, M., S. H. Kim, …, C. Dekker. 2016. Intercalation-Based Single-Molecule Fluorescence Assay To Study DNA Supercoil Dynamics. *Nano Lett.* 16:4699–4707.
8. Kolbeck, P. J., M. Tišma, …, J. Lipfert. 2023. Supercoiling-dependent DNA binding: quantitative modeling and applications to bulk and single-molecule experiments. *Nucleic Acids Res.* 52:59–72.
9. McGann, L. E., and M. L. Walterson. 1987. Cryoprotection by dimethyl sulfoxide and dimethyl sulfone. *Cryobiology.* 24:11–16.
10. Kashino, G., Y. Liu, …, M. Watanabe. 2010. An Alternative Mechanism for Radioprotection by Dimethyl Sulfoxide; Possible Facilitation of DNA Double-strand Break Repair. *J. Radiat. Res.* 51:733–740.
11. Noda, M., Y. Ma, …, K. Yoshikawa. 2017. A single-molecule assessment of the protective effect of DMSO against DNA double-strand breaks induced by photo-and γ-ray-irradiation, and freezing. *Sci. Rep.* 7:8557.
12. Juang, J.-K., and H.-J. Liu. 1987. The effect of DMSO on natural DNA conformation in enhancing transcription. *Biochem. Biophys. Res. Commun.* 146:1458–1464.
13. Winship, P. R. 1989. An lmproved method for directly sequencing PCR-amplified material using dimethyl sulphoxide. *Nucleic Acids Res.* 17:1266.
14. Bachmann, B., W. Lüke, and G. Hunsmann. 1990. Improvement of PCR amplified DNA sequencing with the aid of detergents. *Nucleic Acids Res.* 18:1309.
15. Chester, N., and D. R. Marshak. 1993. Dimethyl sulfoxide-mediated primer Tm reduction: a method for analyzing the role of renaturation temperature in the polymerase chain reaction. *Anal. Biochem.* 209:284–290.
16. Varadaraj, K., and D. M. Skinner. 1994. Denaturants or cosolvents improve the specificity of PCR amplification of a G + C-rich DNA using genetically engineered DNA polymerases. *Gene.* 140:1–5.
17. Frackman, S., G. Kobs, …, D. Storts. 1998. Betaine and DMSO: enhancing agents for PCR. *Promega notes.* 65:27–29.
18. Chakrabarti, R., and C. E. Schutt. 2001. The enhancement of PCR amplification by low molecular-weight sulfones. *Gene.* 274:293–298.
19. Roux, K. H. 2009. Optimization and Troubleshooting in PCR. *Cold Spring Harb. Protoc.* 2009:pdb.ip66.
20. Jensen, M. A., M. Fukushima, and R. W. Davis. 2010. DMSO and Betaine Greatly Improve Amplification of GC-Rich Constructs in De Novo Synthesis. *PLoS One.* 5:e11024.
21. Lv, B., Y. Dai, …, D. Li. 2015. The Effect of Dimethyl Sulfoxide on Supercoiled DNA Relaxation Catalyzed by Type I Topoisomerases. *BioMed Res. Int.* 2015:1–8.
22. Escara, J. F., and J. R. Hutton. 1980. Thermal stability and renaturation of DNA in dimethyl sulfoxide solutions: Acceleration of the renaturation rate. *Biopolymers.* 19:1315–1327.
23. Markarian, S. A., A. M. Asatryan, …, H. R. Sargsyan. 2006. Effect of diethylsulfoxide on the thermal denaturation of DNA. *Biopolymers.* 82:1–5.
24. Nakano, S.-i., and N. Sugimoto. 2016. The structural stability and catalytic activity of DNA and RNA oligonucleotides in the presence of organic solvents. *Biophys. Rev.* 8:11–23.
25. Tunçer, S., R. Gurbanov, …, S. Banerjee. 2018. Low dose dimethyl sulfoxide driven gross molecular changes have the potential to interfere with various cellular processes. *Sci. Rep.* 8:14828.
26. Enlund, E., S. Julin, …, M. A. Kostiainen. 2024. Structural stability of DNA origami nanostructures in organic solvents. *Nanoscale.* 16:13407–13415.
27. Xu, M., T. Dai, …, G. Yang. 2022. The incipient denaturation mechanism of DNA. *RSC Adv.* 12:23356–23365.
28. Garcia, H. G., P. Grayson, …, P. A. Wiggins. 2007. Biological consequences of tightly bent DNA: The other life of a macromolecular celebrity. *Biopolymers.* 85:115–130.
29. Kriegel, F., N. Ermann, and J. Lipfert. 2017. Probing the mechanical properties, conformational changes, and interactions of nucleic acids with magnetic tweezers. *J. Struct. Biol.* 197:26–36.
30. Tian, F. J., C. Zhang, …, L. Dai. 2023. Universality in RNA and DNA deformations induced by salt, temperature change, stretching force, and protein binding. *Proc. Natl. Acad. Sci. USA.* 120:e2218425120.
31. Dong, H.-L., C. Zhang, …, Z.-J. Tan. 2024. The origin of different bending stiffness between double-stranded RNA and DNA revealed by magnetic tweezers and simulations. *Nucleic Acids Res.* 52:2519–2529.
32. Anderson, P., and W. Bauer. 1978. Supercoiling in closed circular DNA: dependence upon ion type and concentration. *Biochemistry.* 17:594–601.
33. Baumann, C. G., S. B. Smith, …, C. Bustamante. 1997. Ionic effects on the elasticity of single DNA molecules. *Proc. Natl. Acad. Sci. USA.* 94:6185–6190.
34. Wenner, J. R., M. C. Williams, …, V. A. Bloomfield. 2002. Salt dependence of the elasticity and overstretching transition of single DNA molecules. *Biophys. J.* 82:3160–3169.
35. Tempestini, A., V. Cassina, …, F. Mantegazza. 2013. Magnetic tweezers measurements of the nanomechanical stability of DNA against denaturation at various conditions of pH and ionic strength. *Nucleic Acids Res.* 41:2009–2019.
36. Lipfert, J., S. Doniach, …, D. Herschlag. 2014. Understanding nucleic acid-ion interactions. *Annu. Rev. Biochem.* 83:813–841.
37. Cruz-León, S., W. Vanderlinden, …, N. Schwierz. 2022. Twisting DNA by salt. *Nucleic Acids Res.* 50:5726–5738.
38. Kriegel, F., N. Ermann, …, J. Lipfert. 2017. Probing the salt dependence of the torsional stiffness of DNA by multiplexed magnetic torque tweezers. *Nucleic Acids Res.* 45:5920–5929.
39. Odijk, T. 1977. Polyelectrolytes Near the Rod Limit. *J. Polym. Sci. Polym. Phys. Ed.* 15:477–483.
40. Skolnick, J., and M. Fixman. 1977. Electrostatic Persistence Length of a Wormlike Polyelectrolyte. *Macromolecules.* 10:944–948.
41. Lipfert, J., G. M. Skinner, …, N. H. Dekker. 2014. Double-stranded RNA under force and torque: Similarities to and striking differences from double-stranded DNA. *Proc. Natl. Acad. Sci. USA.* 111:15408–15413.
42. Ivanov, V. I., L. E. Minchenkova, …, A. I. Poletayev. 1973. Different conformations of double-stranded nucleic acid in solution as revealed by circular dichroism. *Biopolymers.* 12:89–110.
43. Nejedl, K., J. Chládková, …, J. Kypr. 2005. Mapping the B-A conformational transition along plasmid DNA. *Nucleic Acids Res.* 33:e5.
44. Nakano, S.-i., L. Wu, …, N. Sugimoto. 2008. Conformation and the sodium ion condensation on DNA and RNA structures in the presence of a neutral cosolute as a mimic of the intracellular media. *Mol. Biosyst.* 4:579–588.
45. Hormeño, S., F. Moreno-Herrero, …, J. . Arias-Gonzalez. 2011. Condensation Prevails over B-A Transition in the Structure of DNA at Low Humidity. *Biophys. J.* 100:2006–2015.
46. Ma, Y., Y. Yoshikawa, …, K. Yoshikawa. 2020. Marked Difference in the Conformational Transition of DNA Caused by Propanol Isomer. *Polymers.* 12:1607.
47. Del Vecchio, P., D. Esposito, …, G. Barone. 1999. The effects of polyols on the thermal stability of calf thymus DNA. *Int. J. Biol. Macromol.* 24:361–369.
48. Zheng, C.-C., Y.-L. Chen, …, Z.-J. Tan. 2024. Effect of ethanol on the elasticities of double-stranded RNA and DNA revealed by magnetic tweezers and simulations. *J. Chem. Phys.* 161
49. Strick, T. R., J. F. Allemand, …, V. Croquette. 1996. The elasticity of a single supercoiled DNA molecule. *Science.* 271:1835–1837.
50. Vilfan, I. D., J. Lipfert, …, N. H. Dekker. 2009. Magnetic Tweezers for Single-Molecule Experiments. *In* Handbook of Single-Molecule Biophysics. P. Hinterdorfer and A. van Oijen, eds Springer.

51. Lipfert, J., X. Hao, and N. H. Dekker. 2009. Quantitative modeling and optimization of magnetic tweezers. *Biophys. J.* 96:5040–5049.
52. Binnig, G., C. F. Quate, and C. Gerber. 1986. Atomic Force Microscope. *Phys. Rev. Lett.* 56:930–933.
53. Lyubchenko, Y. L., and L. S. Shlyakhtenko. 2009. AFM for analysis of structure and dynamics of DNA and protein–DNA complexes. *Methods.* 47:206–213.
54. Hansma, H. G., D. E. Laney, …, P. K. Hansma. 1995. Applications for atomic force microscopy of DNA. *Biophys. J.* 68:1672–1677.
55. Konrad, S., W. Vanderlinden, and J. Lipfert. 2021. A High-throughput Pipeline to Determine DNA and Nucleosome Conformations by AFM Imaging. *Bio-protocol.* 11:e4180.
56. Savelyev, A., and G. A. Papoian. 2010. Chemically accurate coarse graining of double-stranded DNA. *Proc. Natl. Acad. Sci. USA.* 107:20340–20345.
57. Schöpflin, R., H. Brutzer, …, G. Wedemann. 2012. Probing the Elasticity of DNA on Short Length Scales by Modeling Supercoiling under Tension. *Biophys. J.* 103:323–330.
58. Doye, J. P. K., T. E. Ouldridge, …, W. P. J. Smith. 2013. Coarse-graining DNA for simulations of DNA nanotechnology. *Phys. Chem. Chem. Phys.* 15:20395–20414.
59. Vanderlinden, W., E. Skoruppa, …, J. Lipfert. 2022. DNA fluctuations reveal the size and dynamics of topological domains. *PNAS Nexus.* 1.
60. Ott, K., L. Martini, …, U. Gerland. 2020. Dynamics of the Buckling Transition in Double-Stranded DNA and RNA. *Biophys. J.* 118:1690–1701.
61. de Jager, M., P. J. Kolbeck, …, L. Filion. 2024. Exploring protein-mediated compaction of DNA by coarse-grained simulations and unsupervised learning. *Biophys. J.* 123:3231–3241.
62. Sun, T., V. Minhas, …, L. Nordenskiöld. 2021. Bottom-Up Coarse-Grained Modeling of DNA. *Front. Mol. Biosci.* 8
63. Popov, Y. O., and A. V. Tkachenko. 2005. Effects of kinks on DNA elasticity. *Phys. Rev. E.* 71:051905.
64. Wiggins, P. A., R. Phillips, and P. C. Nelson. 2005. Exact theory of kinkable elastic polymers. *Phys. Rev. E.* 71:021909.
65. Lipfert, J., D. A. Koster, …, N. H. Dekker. 2009. Single-molecule magnetic tweezers studies of type IB topoisomerases. *Methods Mol. Biol.* 582:71–89.
66. Walker, P. U., W. Vanderlinden, and J. Lipfert. 2018. Dynamics and energy landscape of DNA plectoneme nucleation. *Phys. Rev. E.* 98:042412.
67. Kolbeck, P. J., W. Vanderlinden, …, J. Lipfert. 2021. Molecular structure, DNA binding mode, photophysical properties and recommendations for use of SYBR Gold. *Nucleic Acids Res.* 49:5143–5158.
68. van Loenhout, M. J., J. W. J. Kerssemakers, …, C. Dekker. 2012. Non-bias-limited tracking of spherical particles, enabling nanometer resolution at low magnification. *Biophys. J.* 102:2362–2371.
69. Cnossen, J. P., D. Dulin, and N. H. Dekker. 2014. An optimized software framework for real-time, high-throughput tracking of spherical beads. *Rev. Sci. Instrum.* 85:103712.
70. Strick, T. R., V. Croquette, and D. Bensimon. 1998. Homologous pairing in stretched supercoiled DNA. *Proc. Natl. Acad. Sci. USA.* 95:10579–10583.
71. Vanderlinden, W., J. Lipfert, …, S. De Feyter. 2014. Structure, mechanics, and binding mode heterogeneity of LEDGF/p75-DNA nucleoprotein complexes revealed by scanning force microscopy. *Nanoscale.* 6:4611–4619.
72. Konrad, S. F., W. Vanderlinden, …, J. Lipfert. 2021. High-throughput AFM analysis reveals unwrapping pathways of H3 and CENP-A nucleosomes. *Nanoscale.* 13:5435–5447.
73. Phillips, R., J. Kondev, …, H. Garcia. 2013. Physical Biology of the Cell. 2nd Edition. *Garland Science*. https://doi.org/10.1201/9781134111589.
74. Sakai T. 2020. Single Polymer Chain. In: Sakai T., editor. Physics of Polymer Gels; Wiley-VCH Verlag GmbH & Co. KGaA 2020. p. 1–22.
75. Rivetti, C., M. Guthold, and C. Bustamante. 1996. Scanning force microscopy of DNA deposited onto mica: equilibration versus kinetic trapping studied by statistical polymer chain analysis. *J. Mol. Biol.* 264:919–932.
76. Brouns, T., H. De Keersmaecker, …, W. Vanderlinden. 2018. Free Energy Landscape and Dynamics of Supercoiled DNA by High-Speed Atomic Force Microscopy. *ACS Nano.* 12:11907–11916.
77. Abels, J. A., F. Moreno-Herrero, …, N. H. Dekker. 2005. Single-molecule measurements of the persistence length of double-stranded RNA. *Biophys. J.* 88:2737–2744.
78. Nomidis, S. K., E. Skoruppa, …, J. F. Marko. 2019. Twist-bend coupling and the statistical mechanics of the twistable wormlike-chain model of DNA: Perturbation theory and beyond. *Phys. Rev. E.* 99:032414.
79. Skoruppa, E., and E. Carlon. 2022. Equilibrium fluctuations of DNA plectonemes. *Phys. Rev. E.* 106:024412.
80. Skoruppa, E., and H. Schiessel. 2025. Systematic coarse-graining of sequence-dependent structure and elasticity of double-stranded DNA. *Phys. Rev. Res.* 7:013044.
81. Olson, W. K., D. Swigon, …, B. D. Coleman. 2004. Implications of the dependence of the elastic properties of DNA on nucleotide sequence. *Philos. Trans. R. Soc. London, Ser. A: Math. Phys. Eng. Sci.* 362:1403–1422.
82. Lankaš, F., O. Gonzalez, …, J. H. Maddocks. 2009. On the parameterization of rigid base and basepair models of DNA from molecular dynamics simulations. *Phys. Chem. Chem. Phys.* 11:10565–10588.
83. Eslami-Mossallam, B., R. D. Schram, …, H. Schiessel. 2016. Multiplexing Genetic and Nucleosome Positioning Codes: A Computational Approach. *PLoS One.* 11:e0156905.
84. Watson, J. D., and F. H. Crick. 1953. Molecular structure of nucleic acids. *Nature.* 171:737–738.
85. Drew, H. R., S. Samson, and R. E. Dickerson. 1982. Structure of a B-DNA dodecamer at 16 K. *Proc. Natl. Acad. Sci. USA.* 79:4040–4044.
86. Murphy, M. C., I. Rasnik, …, T. Ha. 2004. Probing single-stranded DNA conformational flexibility using fluorescence spectroscopy. *Biophys. J.* 86:2530–2537.
87. Sim, A. Y. L., J. Lipfert, …, S. Doniach. 2012. Salt dependence of the radius of gyration and flexibility of single-stranded DNA in solution probed by small-angle x-ray scattering. *Phys. Rev. E.* 86:021901.
88. Kahn, J. D., E. Yun, and D. M. Crothers. 1994. Detection of localized DNA flexibility. *Nature.* 368:163–166.
89. Bustamante, C., J. F. Marko, …, S. Smith. 1994. Entropic elasticity of lambda-phage DNA. *Science.* 265:1599–1600.
90. Marko, J. F., and E. D. Siggia. 1995. Stretching DNA. *Macromolecules.* 28:8759–8770.
91. Bouchiat, C., M. D. Wang, …, V. Croquette. 1999. Estimating the persistence length of a worm-like chain molecule from force-extension measurements. *Biophys. J.* 76:409–413.
92. Lipfert, J., S. Klijnhout, and N. H. Dekker. 2010. Torsional sensing of small-molecule binding using magnetic tweezers. *Nucleic Acids Res.* 38:7122–7132.
93. Chen, L., Y. Wang, and G. Yang. 2023. Locally Denatured DNA Compaction by Divalent Cations. *J. Phys. Chem. B.* 127:4783–4789.
94. Kriegel, F., C. Matek, …, J. Lipfert. 2018. The temperature dependence of the helical twist of DNA. *Nucleic Acids Res.* 46:7998–8009.
95. Bryant, Z., M. D. Stone, …, C. Bustamante. 2003. Structural transitions and elasticity from torque measurements on DNA. *Nature.* 424:338–341.
96. van Mameren, J., P. Gross, …, E. J. G. Peterman. 2009. Unraveling the structure of DNA during overstretching by using multicolor, single-molecule fluorescence imaging. *Proc. Natl. Acad. Sci. USA.* 106:18231–18236.

97. Strick, T., J. F. Allemand, …, D. Bensimon. 2000. Twisting and stretching single DNA molecules. *Prog. Biophys. Mol. Biol.* 74:115–140.

98. Marko, J. F. 2007. Torque and dynamics of linking number relaxation in stretched supercoiled DNA. *Phys. Rev. E.* 76:021926.

99. Wang, X., H. J. Lim, and A. Son. 2014. Characterization of denaturation and renaturation of DNA for DNA hybridization. *Environ. Health Toxicol.* 29:e2014007.

100. Lyubchenko, Y. L., and L. S. Shlyakhtenko. 1997. Visualization of supercoiled DNA with atomic force microscopy in situ. *Proc. Natl. Acad. Sci. USA.* 94:496–501.

101. Bussiek, M., N. Mucke, and J. Langowski. 2003. Polylysine-coated mica can be used to observe systematic changes in the supercoiled DNA conformation by scanning force microscopy in solution. *Nucleic Acids Res.* 31:e137.

102. Kim, S. H., M. Ganji, …, C. Dekker. 2018. DNA sequence encodes the position of DNA supercoils. *eLife.* 7:e36557.

103. Ganji, M., I. A. Shaltiel, …, C. Dekker. 2018. Real-time imaging of DNA loop extrusion by condensin. *Science.* 360:102–105.

104. Kim, E., J. Kerssemakers, …, C. Dekker. 2020. DNA-loop extruding condensin complexes can traverse one another. *Nature.* 579:438–442.

105. Martin-Gonzalez, A., M. Tišma, …, C. Dekker. 2024. DNA supercoiling enhances DNA condensation by ParB proteins. *Nucleic Acids Res.* 52:13255–13268.

106. Cutts, E. E., D. Tetiker, …, L. Aragon. 2024. Molecular mechanism of condensin I activation by KIF4A. *EMBO J.* 44:682–704.

Biophysical Journal, Volume *124*

Supplemental information

The effects of DMSO on DNA conformations and mechanics

Koen R. Storm, Caroline Körösy, Enrico Skoruppa, Stefanie D. Pritzl, Pauline J. Kolbeck, Willem Vanderlinden, Helmut Schiessel, and Jan Lipfert

SUPPLEMENTARY INFORMATION FOR

The Effects of DMSO on DNA Conformations and Mechanics

Koen R. Storm[1,4,†], Caroline Körösy[1,†], Enrico Skoruppa[2], Stefanie D. Pritzl[1], Pauline J. Kolbeck[1], Willem Vanderlinden[3], Helmut Schiessel[2], Jan Lipfert[1,4,*]

[1]Soft Condensed Matter and Biophysics, Department of Physics and Debye Institute for Nanomaterials Science, Utrecht University, Princetonplein 1, 3584 CC Utrecht, The Netherlands

[2]Cluster of Excellence Physics of Life, TU Dresden, Arnoldstrasse 18, 01307 Dresden, Germany

[3]School of Physics and Astronomy, University of Edinburgh, Scotland UK

[4]Institute for Physics, University of Augsburg, Universitätsstrasse 1, 86159 Augsburg, Germany

[†]Equal contribution

[*]Correspondence: J.Lipfert@uu.nl

<u>Content</u>

Supplementary Table S1

Supplementary Figures S1 – S16

Supplementary Table

Supplementary Table S1. Statistical tests comparing DNA end-to-end distance distributions under DMSO-treatment. Results of two-sample two-tailed *t*-tests comparing the means and of two-sample Kolmogorov-Smirnov tests comparing the full distributions of end-to-end distances of DMSO treated DNA samples with the PBS only condition.

2-sample *t*-tests:	
PBS vs. 2% DMSO	$p = 8.3 \times 10^{-6}$
PBS vs. 10% DMSO	$p = 1.7 \times 10^{-2}$
PBS vs. 20% DMSO	$p = 2.4 \times 10^{-12}$
PBS vs. 50% DMSO	$p = 3.7 \times 10^{-18}$

Kolmogorov-Smirnov tests:	
PBS vs. 2% DMSO	$p = 7.8 \times 10^{-6}$
PBS vs. 10% DMSO	$p = 1.2 \times 10^{-3}$
PBS vs. 20% DMSO	$p = 1.6 \times 10^{-11}$
PBS vs. 50% DMSO	$p = 7.0 \times 10^{-16}$

Supplementary Figures

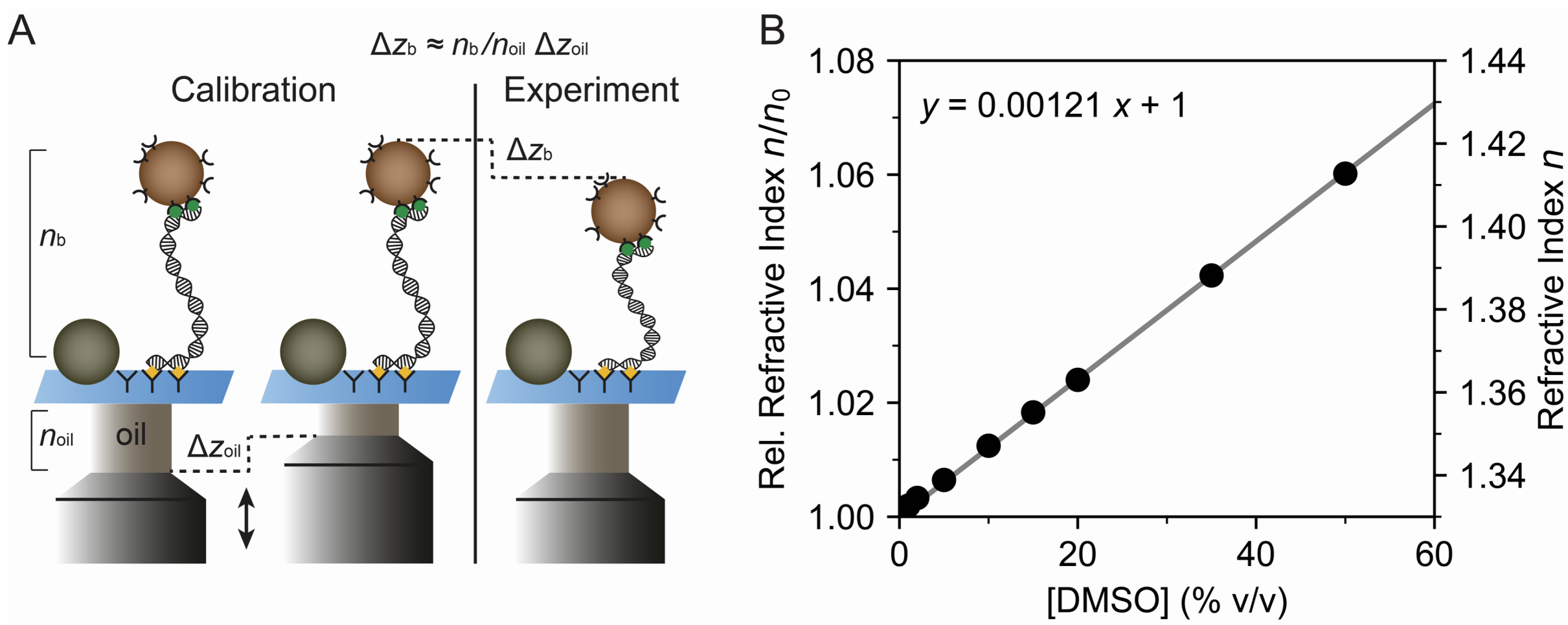

Supplementary Figure S1. Effect of refractive indices on magnetic tweezers measurements. A) Schematic of the determination of vertical displacement using magnetic tweezers. Prior to the experiment, a calibration routine is performed in which diffraction patterns are collected while displacing the oil immersion objective vertically, i.e., a displacement of Δz_{oil}, to construct a so-called look-up table (LUT). During the experiment, the vertical position of the bead is determined by correlation of the diffraction pattern with the previously recorded LUT. To obtain the change in the vertical position of the bead Δz_{b} from the changes determined by comparison with the LUT, one needs to correct for the difference in index of refraction between oil and buffer. The correction factor is, at least approximately, given by the ratio of the refractive index of the oil $n_{\mathrm{oil}} = 1.5154$ and of the buffer n_{b} and we, therefore, determine the absolute Δz_{b} according to $\Delta z_{\mathrm{b}} \approx n_{\mathrm{b}}/n_{\mathrm{oil}} \cdot \Delta z_{\mathrm{oil}}$. B) Refractive index of DMSO solutions in PBS. The solid line is a linear fit to the data relative to the refractive index of water (coefficient of determination $R^2 = 0.999$). For measurements in varying concentrations of DMSO, we still employed a LUT measured using PBS only buffer and we take into account the index of refraction of the (DMSO containing) buffer by using the measured values for different DMSO concentrations for n_{b}.

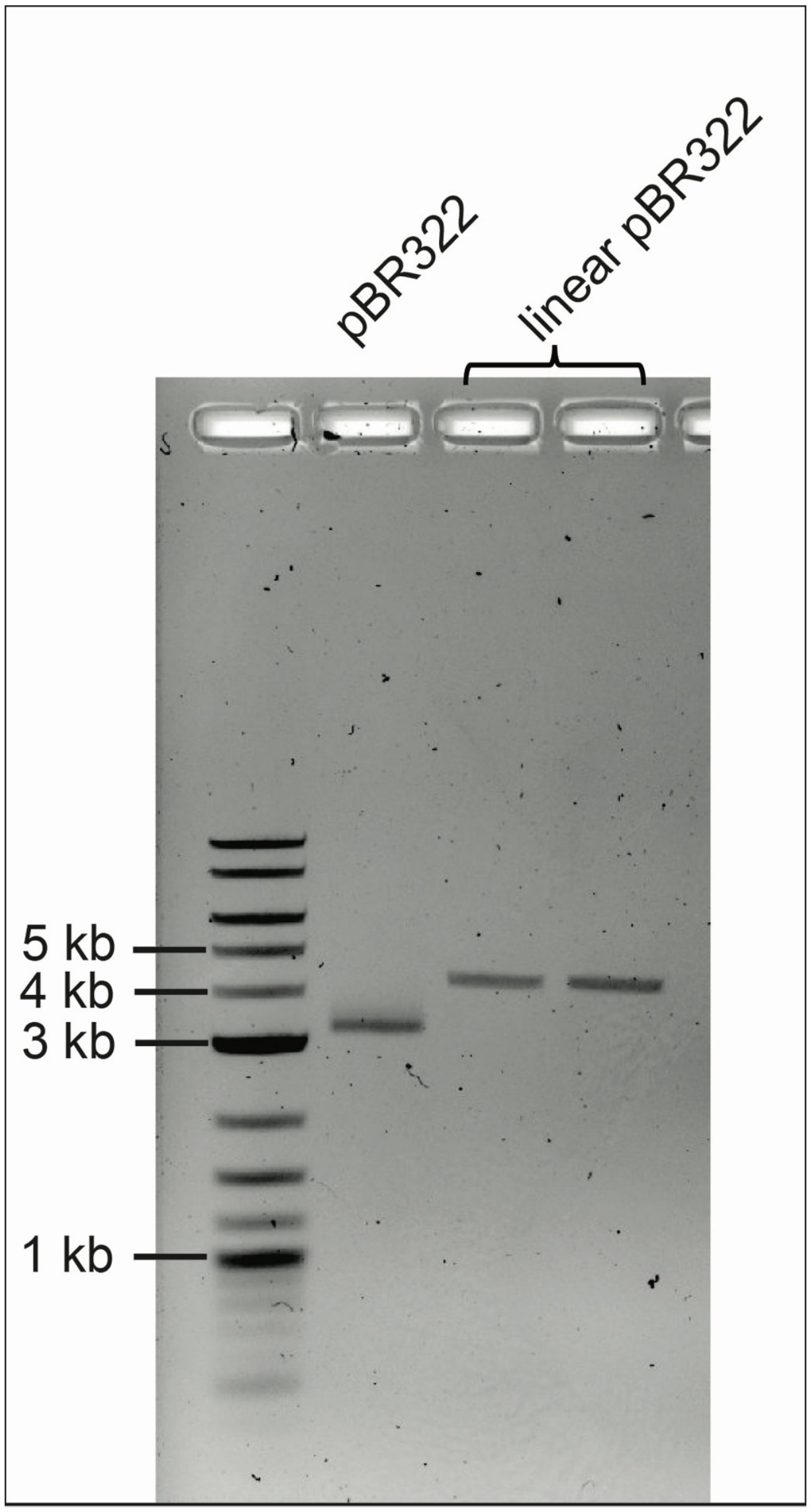

Supplementary Figure S2. Agarose gel image of pBR322 plasmid DNA (1st lane) and two independent linearization batches of pBR322 (2nd and 3rd lane) after linearization with EcoRI at one restriction site in the plasmid. DNA is stained with ROTI-Gel stain and visualized under UV light. 1 kb Plus DNA Ladder (NEB) is used as a size reference, and relevant bands are annotated. Due to the supercoiled structure, the plasmid DNA travels faster through the gel than the linearized DNA. The two independent linearization batches show excellent reproducibility, travel at the expected DNA length (4361 bp for pBR322) compared to the DNA ladder, and run as a single band, suggesting a successful and complete digest.

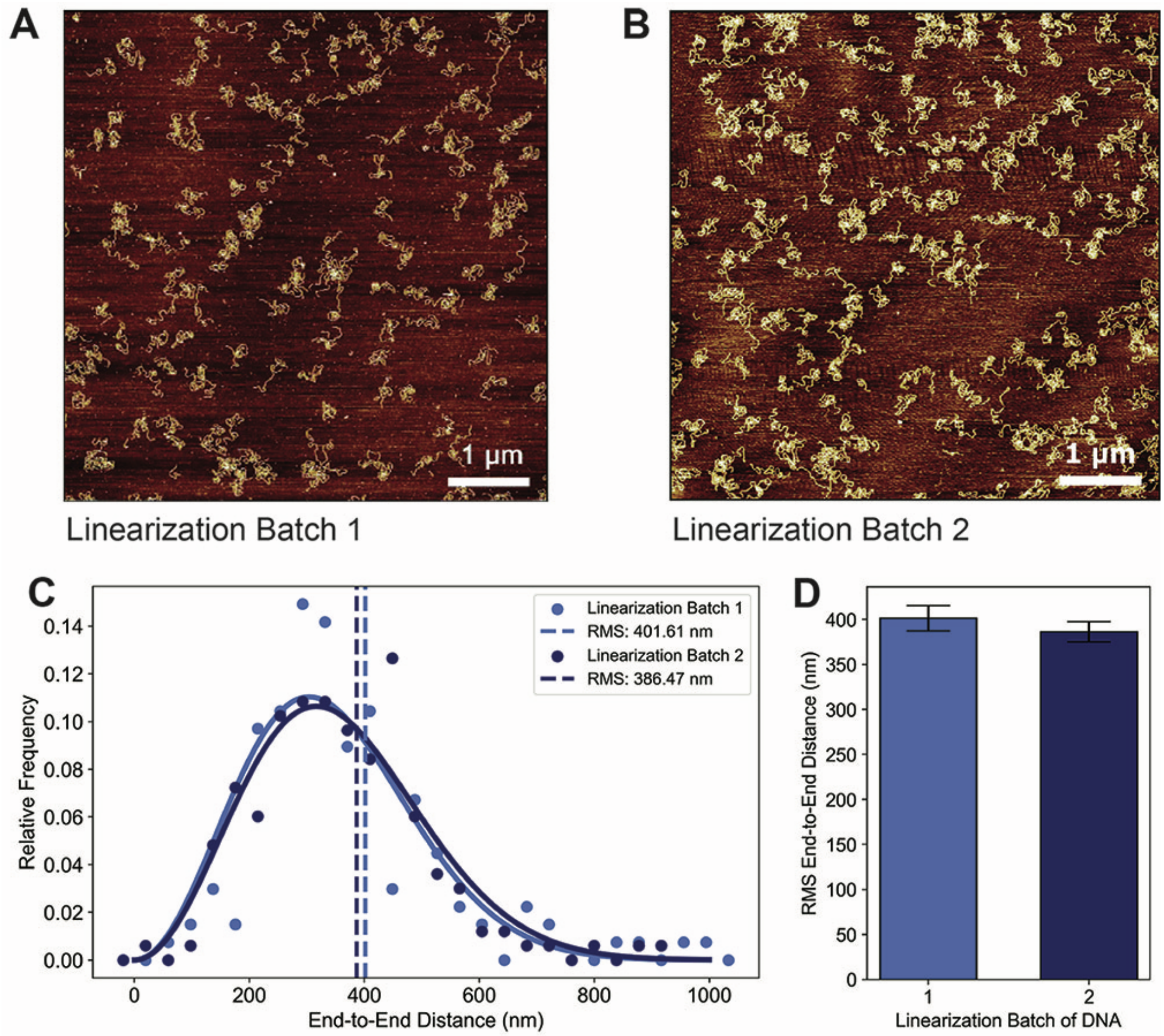

Supplementary Figure S3. Comparison of two independent linearization batches of pBR322. A, B) AMF images of linear DNA (4361 bp) incubated in PBS buffer and imaged on PLL-mica in air. The two DNA batches were prepared in independent linearization reactions (Materials and Methods) and show similar behavior when deposited on the surface. C) Histograms of DNA end-to-end distances in PBS show excellent agreement of distributions for two independent linearization batches. RMS values are visualized as dashed vertical lines. Solid lines are fits of the Gaussian chain model (Equation 1). D) Bar plot comparing the RMS end-to-end distances in PBS for two independent linearization batches of DNA. There is no statistically significant difference between the two batches, neither with regards to the RMS end-to-end distances (mean ± standard deviation) of 402 nm ± 14 nm and 386 nm ± 11 nm, respectively, (the difference was assessed with a two-sample two-tailed *t*-test, $p = 0.6$), nor with regards to the overall distributions (as assessed by a two-sample Kolmogorov-Smirnov test, $p = 0.8$).

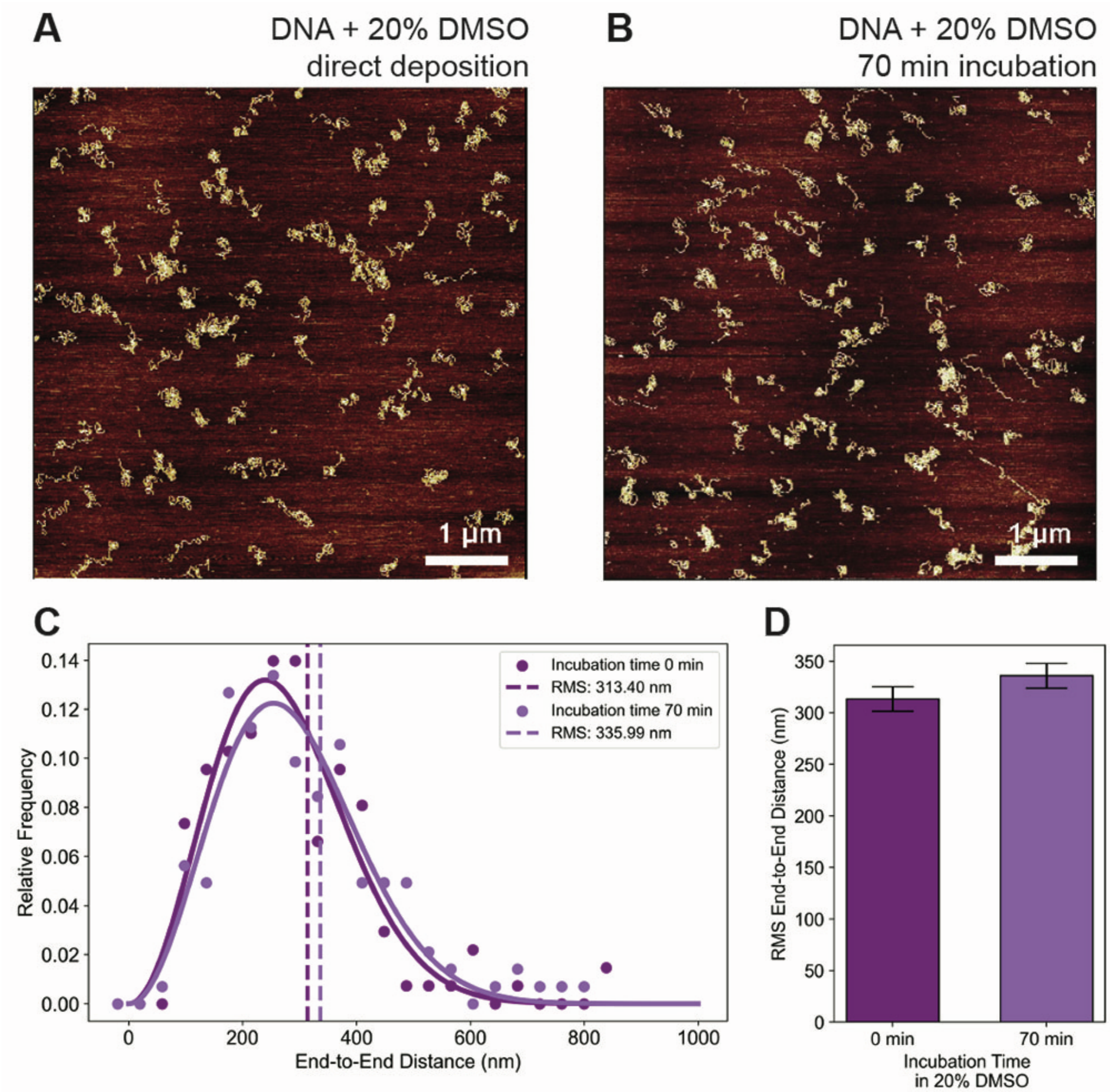

Supplementary Figure S4. Comparison of different incubation times of linear DNA with 20% DMSO before surface deposition. A, B) AMF images of linear DNA (4361 bp) in the 20% (v/v) DMSO condition on PLL-mica measured in air. The two batches of DNA were prepared either by incubating briefly (~30 s, A) or for a much longer time (70 min, B) in the final DMSO concentration prior surface deposition. C) Histograms of DNA end-to-end distances in 20% DMSO with the short (dark purple) and long (light purple) incubation time. The RMS values of 313 ± 12 nm and 336 ± 12 nm are visualized as dashed vertical lines. Solid lines are fits of the Gaussian chain model (Equation 1). D) Bar plot comparing the RMS end-to-end distances in 20% DMSO with and without incubation time before depositing the sample. There is no statistically significant difference between the batches with the short and long incubation time, neither with regards to the RMS end-to-end distances (as assessed with a two-sample two-tailed *t*-test, $p = 0.2$) nor with regards to the overall distributions (as assessed by a two-sample Kolmogorov-Smirnov test, $p = 0.3$).

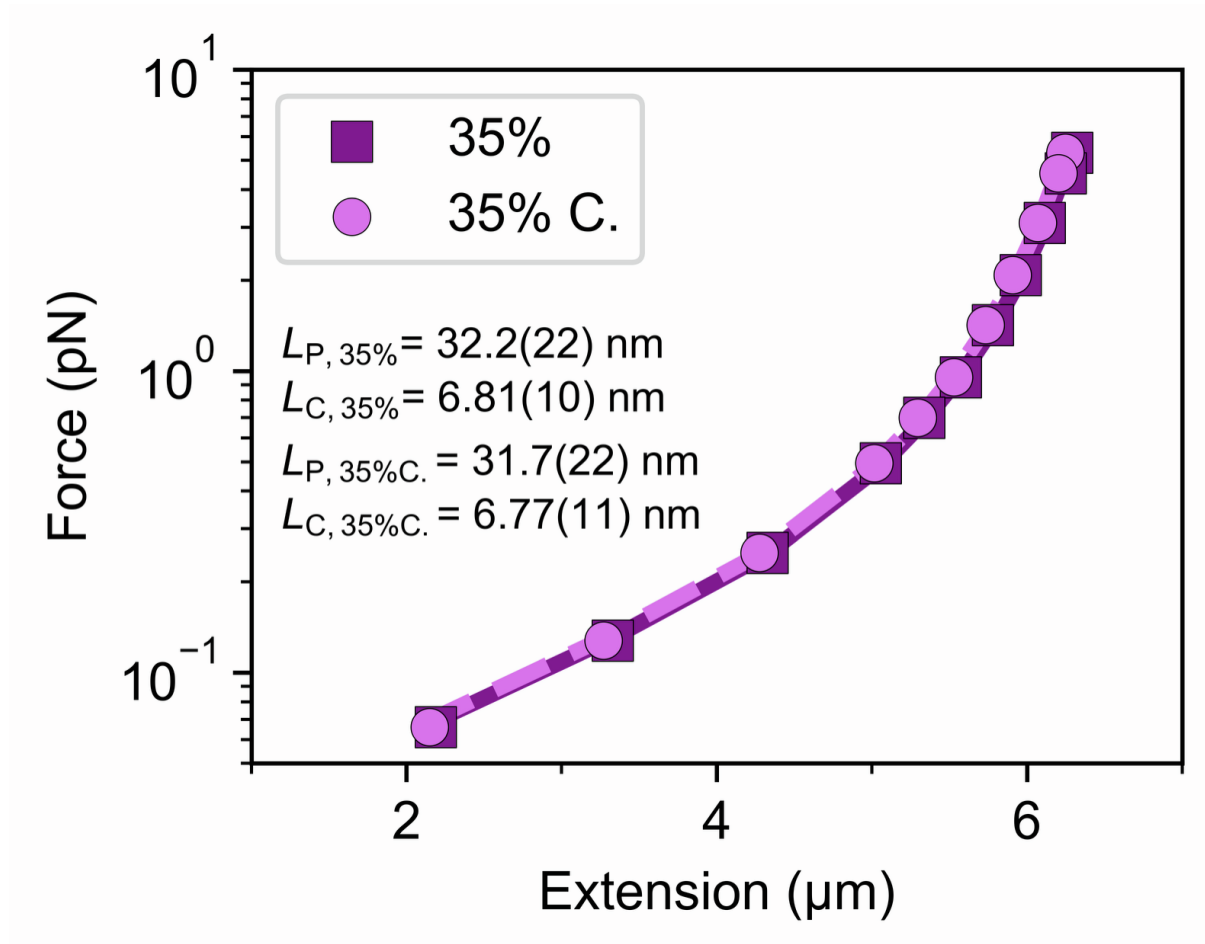

Supplementary Figure S5. Control measurements adjusting the ionic strength after addition of DMSO. Force-extension curves for 20.6 kbp DNA, similar to the data shown in Figure 1, for a DMSO concentration of 35%. For the data labeled "35%", 35% DMSO were added to 1x PBS buffer, as was done for the other DMSO concentrations used in the main text of the paper. For the control data labeled "35% C.", the initial PBS concentration was increased, such that it corresponds to 1x PBS after addition of 35% DMSO. Both samples give essentially identical force-extension curves and the fitted values of the contour and bending persistence lengths are within experimental error (the values in the parentheses indicate the standard deviation of 11 independent molecules as the last significant digit).

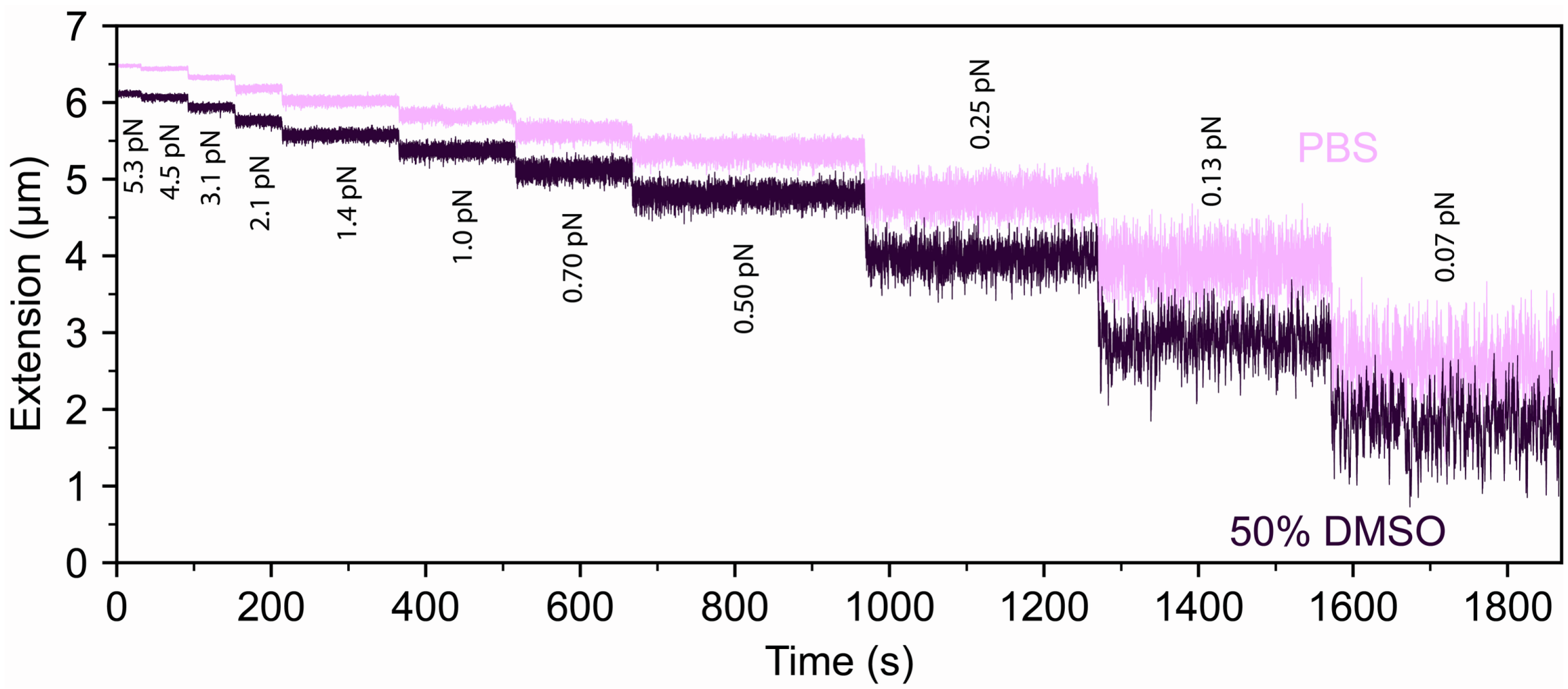

Supplementary Figure S6. Extension-time traces of DNA in PBS buffer and in the presence of 50% DMSO. Extension-time traces recorded for the same 20.6 kbp DNA molecule in PBS buffer only (light magenta) and after adding 50% DMSO (dark red). Forces are indicated in the figure. For both conditions the molecule's extension shortens and extension fluctuations increase with decreasing force, due to its entropic stretching elasticity. Overall, the data for PBS only and 50% DMSO look similar. The 50% DMSO conditions has a reduced tether extension at each force (as seen also in Figure 1B). Importantly, unlike at 60% DMSO, no sudden collapse or dramatic change in DNA behavior is observed.

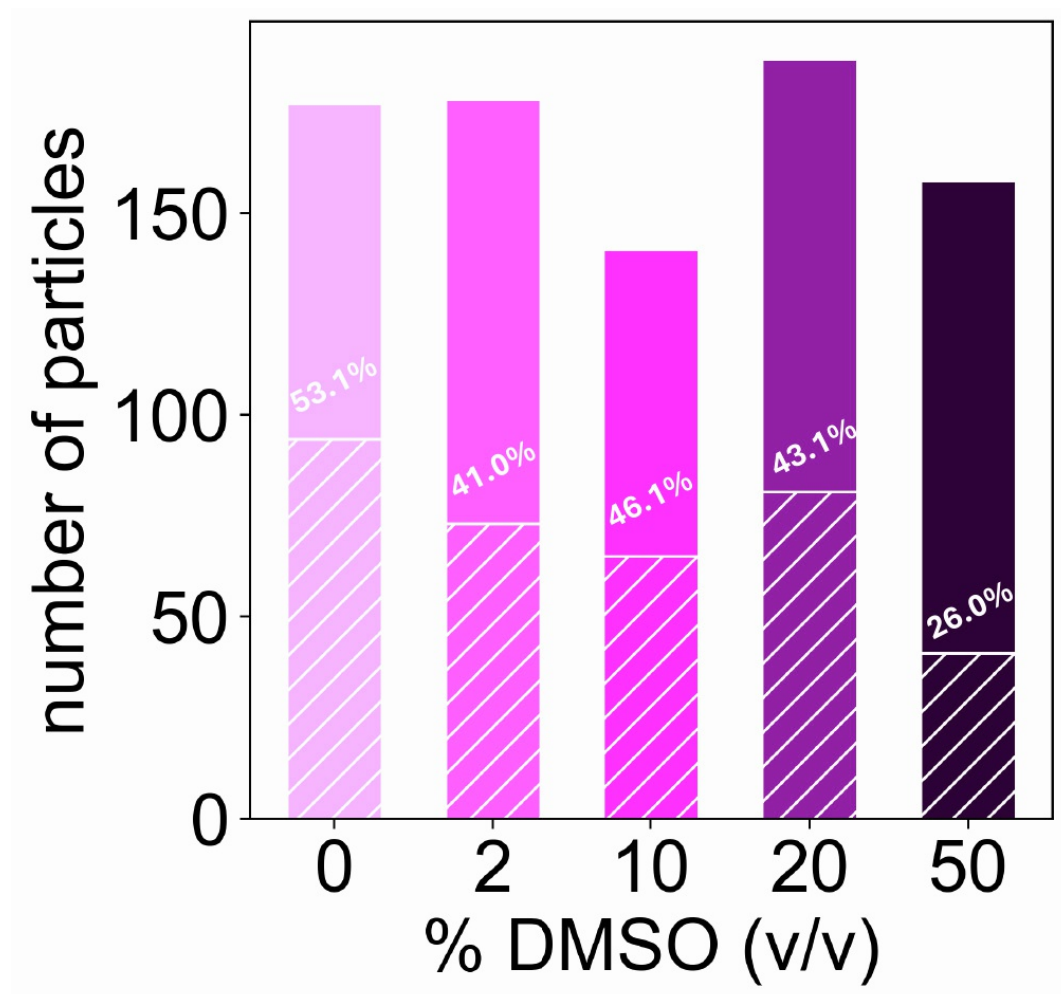

Supplementary Figure S7. Number of molecules analyzed for manual shape analysis and fraction of molecules for end-to-end distance analysis in varying DMSO concentrations. Colored boxes (blue to purple with increasing DMSO) indicate the number of single molecules used for manual shape analysis, the striped boxes indicate the number of the single molecules with visible free ends for end-to-end distance analysis. The data shown are from analysis of all single molecules from 3 images per condition; the end-to-end distance data in Figure 5 uses additional images with similar percentages. We can measure the end-to-end distances for approximately half of the single molecules but are limited to ~25% in 50% DMSO where we observe a more substantial compaction of DNA molecules.

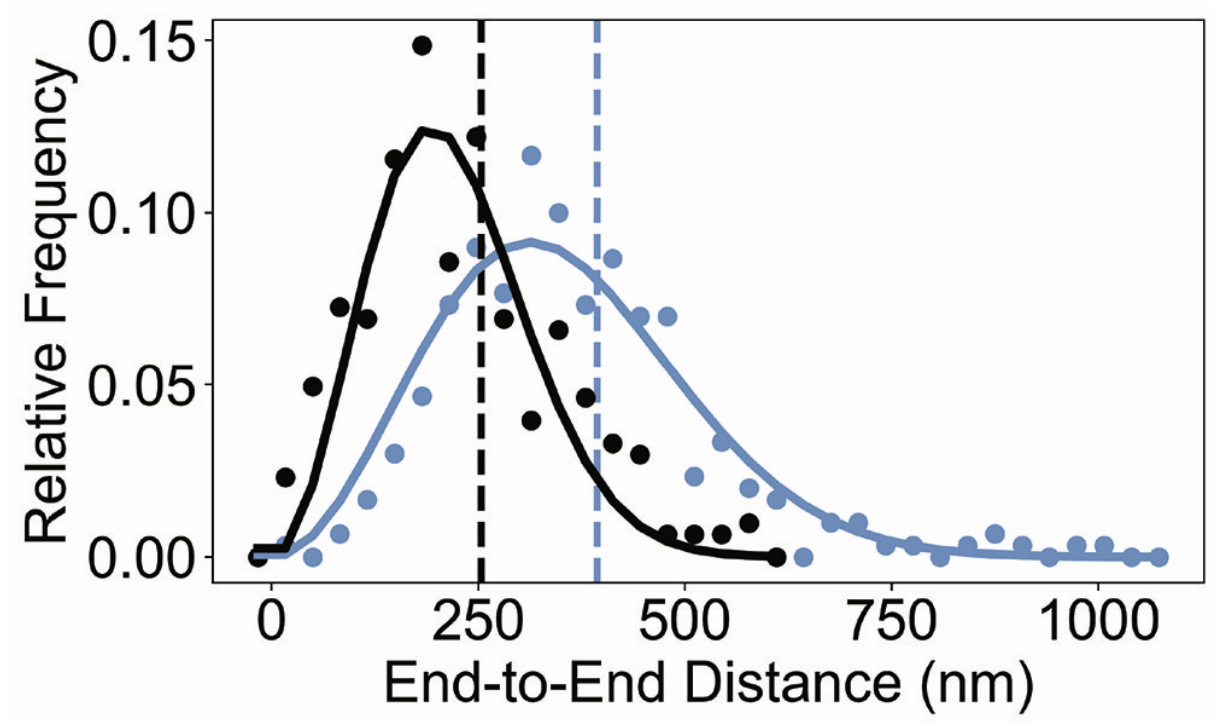

Supplementary Figure S8. Comparison of DNA end-to-end distance distribution for linearized pBR322 plasmid DNA in PBS buffer and in buffer containing 5 mM Mg^{2+}. Blue data points are the data in PBS buffer, from this work (Materials and Methods). The black data points are the data from Brouns *et al*. (ACS Nano, 12(12):11907-11916, 2018; Ref. 49 of the main text). Both data sets were obtained by AFM imaging in air of linearized DNA (4361 bp) deposited on a PLL-coated mica surfaces. Overall, the distributions of end-to-end distances look similar. However, the data in the 5 mM Mg^{2+} conditions show smaller mean end-to-end distances (253 ± 7 nm, black vertical dashed line) in comparison with our results (393 ± 8 nm, blue vertical dashed line) , which is to be expected since Mg^{2+} reduces the bending persistence length of DNA and tends to compact DNA. The lines are fits of the Gaussian chain model (Equation 1) to the data.

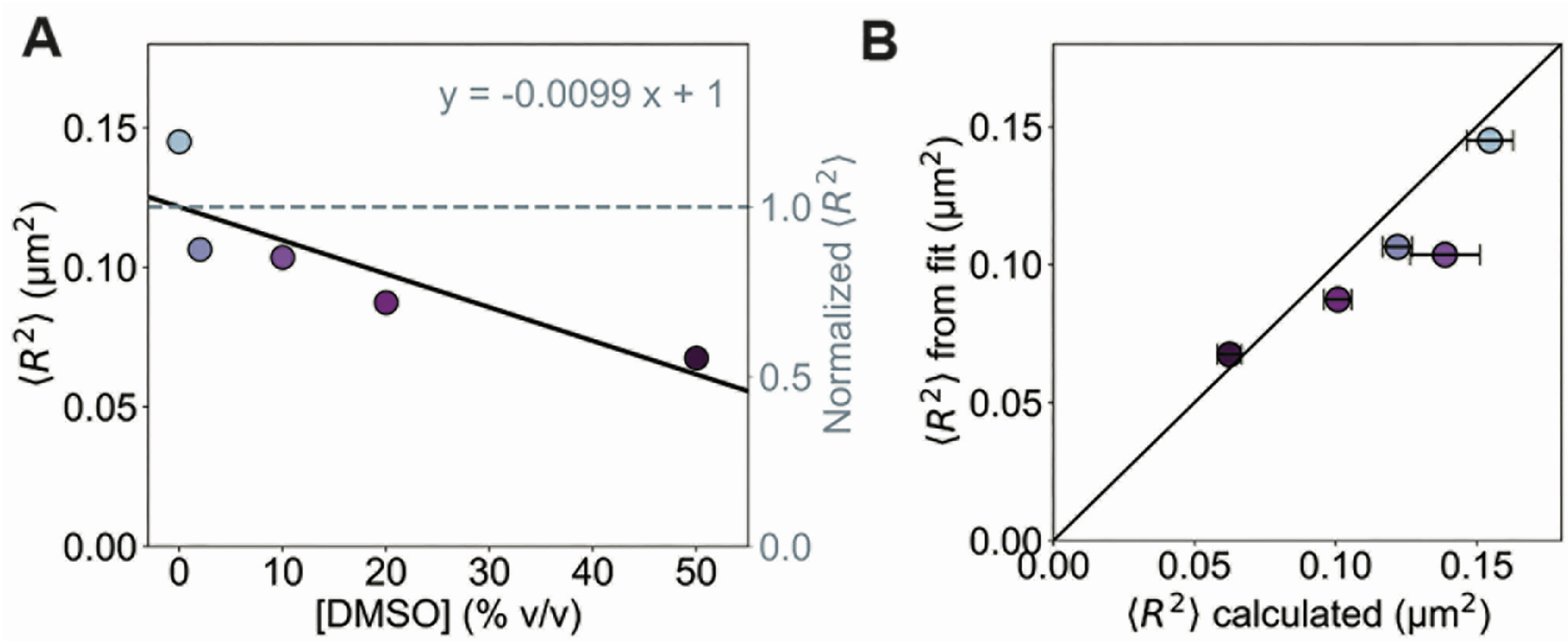

Supplementary Figure S9. Comparison of calculated and fitted <R^2> . A) <R^2> values obtained from fits of the Gaussian chain model (Equation 1 in Materials and Methods) as a function of DMSO concentration. The straight line indicates a linear fit corresponding to ~1% decrease in <R^2> per %-DMSO. This figure is similar to Figure 5C, where we show <R^2> values directly calculated from the end-to-end distances measured in AFM images (and find a change of 1.15% per %-DMSO). B) Correlation of <R^2> values from the direct calculation and from the Gaussian model fits. Overall, the two approaches to obtaining <R^2> are in close agreement. The fitted values show a tendency to smaller values compared to the directly computed <R^2> values. The black line is a 45-degree line as a guide to the eye.

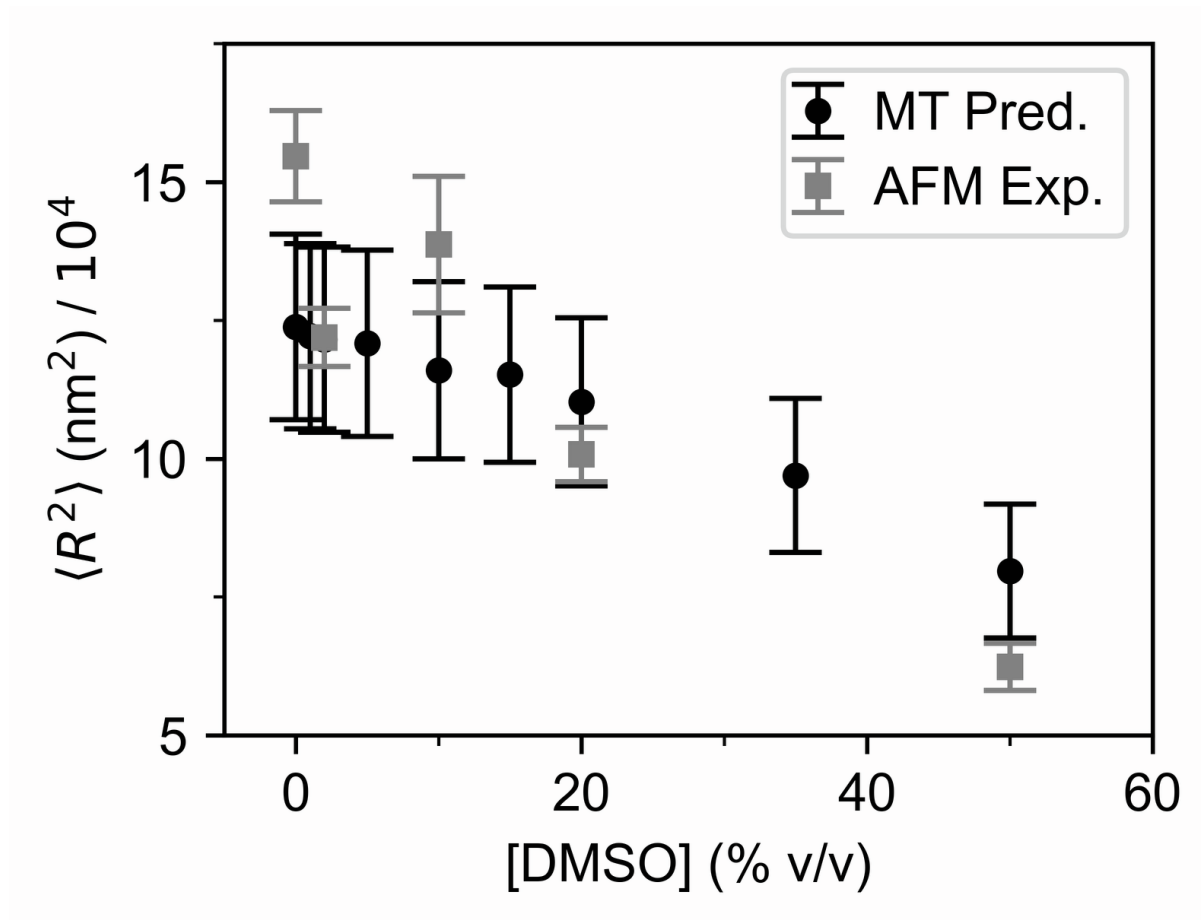

Supplementary Figure S10. Comparison of measured DNA end-to-end distances by AFM imaging and predictions based on parameters from MT force spectroscopy. Grey squares are the $<R^2>$ values measured experimentally by AFM imaging in air after deposition in PLL-mica (Materials and Methods; Figures 3 and 4). Black circles are predictions of the 3D mean squared end-to-end distance from Equation 1 using the values for the bending persistence length L_P and contour length L_C parameters determined by MT force spectroscopy (Figure 1). Error bars for the black data points are from the propagation of experimental uncertainties of the measured parameters.

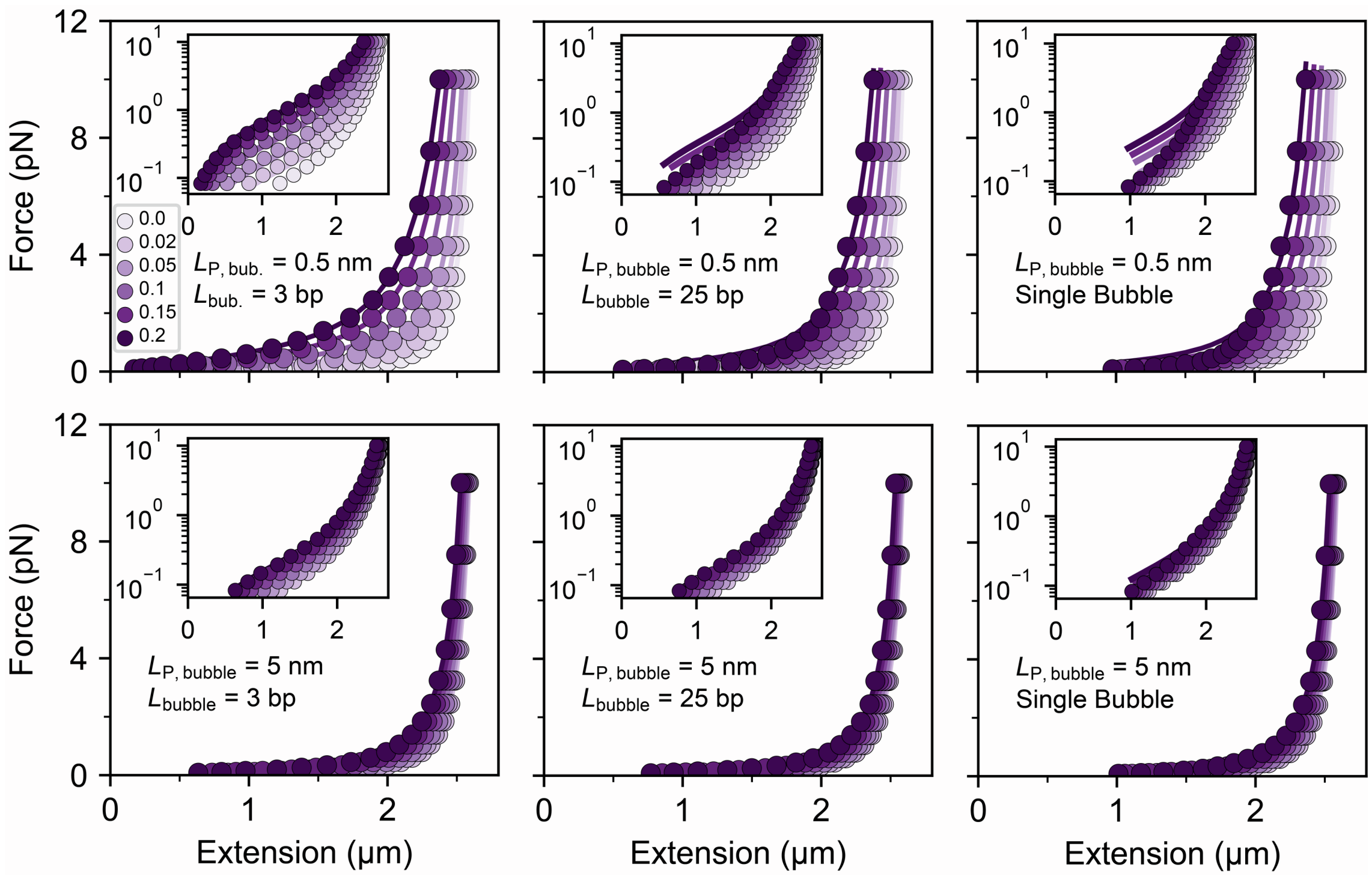

Supplementary Figure S11. Force-extension relationships for coarse-grained DNA simulations with bubbles. Force-extension relationships from coarse-grained Monte Carlo simulations (symbols) and fits of the WLC model (solid lines). The different colors in each panel indicate different bubble fractions f_{bubble} (increasing from light to dark shades; see legend in the top left panel; the same color code applies to all panels). The simulations systematically varied the flexibility of the bubble segments $L_{\text{P,bubble}}$ in the range 0.5 nm to 5 nm. Shown here are the data for $L_{\text{P,bubble}}$ = 0.5 nm (top row) and 5 nm (bottom row). In addition, we systematically varied the size of the flexible bubble regions, L_{bubble}, in the range of 3 bp to 10,000 bp. The 10,000 bp case corresponds to the situation that all bubble segments are in one cluster, i.e. that there is a single bubble that comprises all flexible segments. Shown are the data for L_{bubble} = 3 bp (left panels), 25 bp (middle panels), and the single bubble case (right panels). The insets show the same data in log-linear representation.

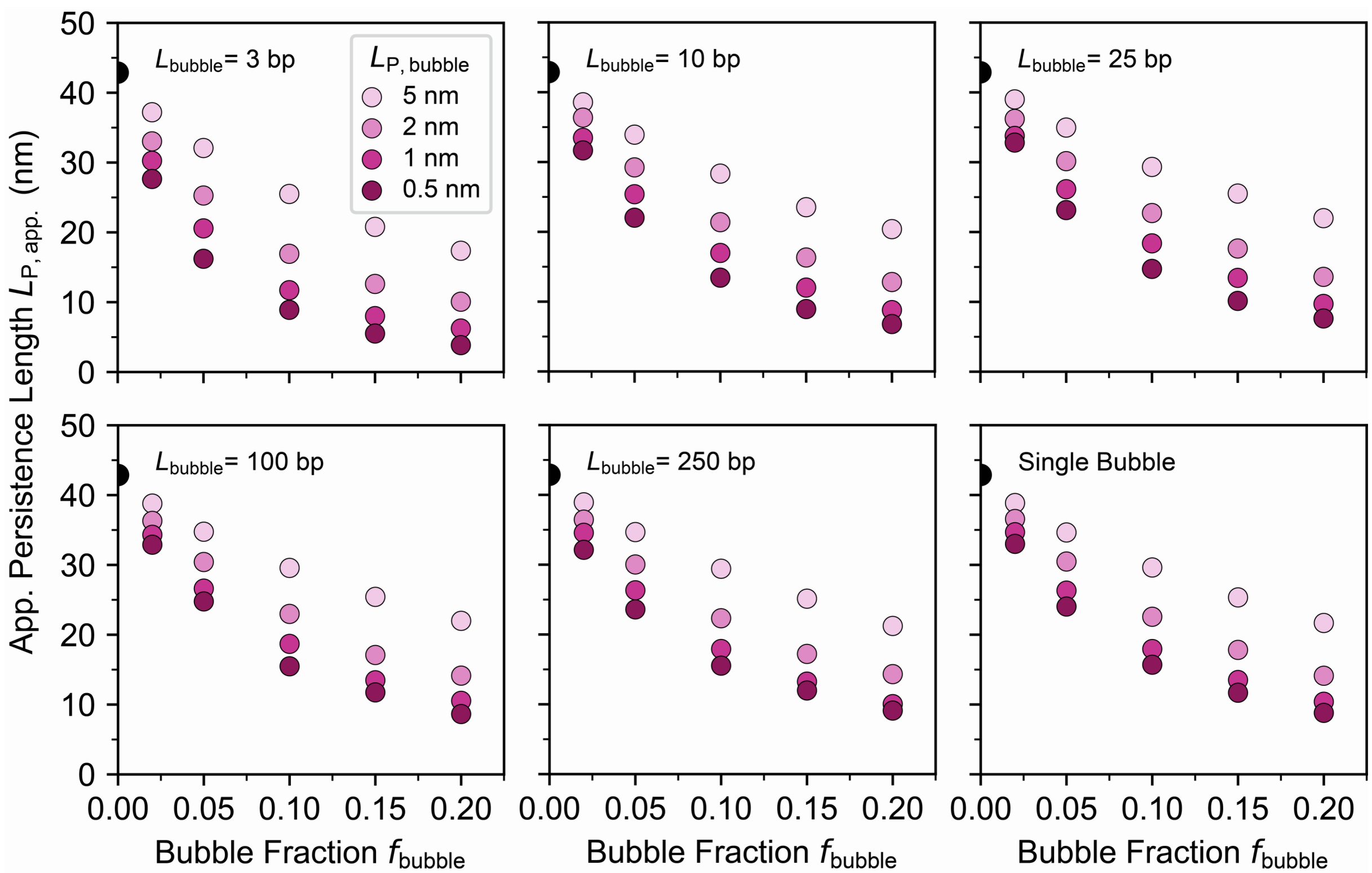

Supplementary Figure S12. Apparent bending persistence lengths from coarse-grained DNA simulations with bubbles. Values for the apparent bending persistence length obtained from WLC fits to the simulated force-extension responses of DNA with flexible bubbles (examples of the data and fits are shown in Supplementary Figure S10). Values for the fitted apparent L_P are shown as a function of the bubble fraction and stiffness of the bubbles $L_{P,bubble}$ (color coded from dark to light shades; see legend in the top left panel; the same color code is used for all panels). The different panels show data for different bubble sizes L_{bubble}. The "single bubble" condition has L_{bubble} = 10,000 bp, such that all bubble segments are in one cluster. The black dots at a bubble fraction of 0 corresponds to the value determined from coarse-grained simulations of the discrete WLC without flexible bubbles, which accurately recover the input bending persistent length of 43 nm.

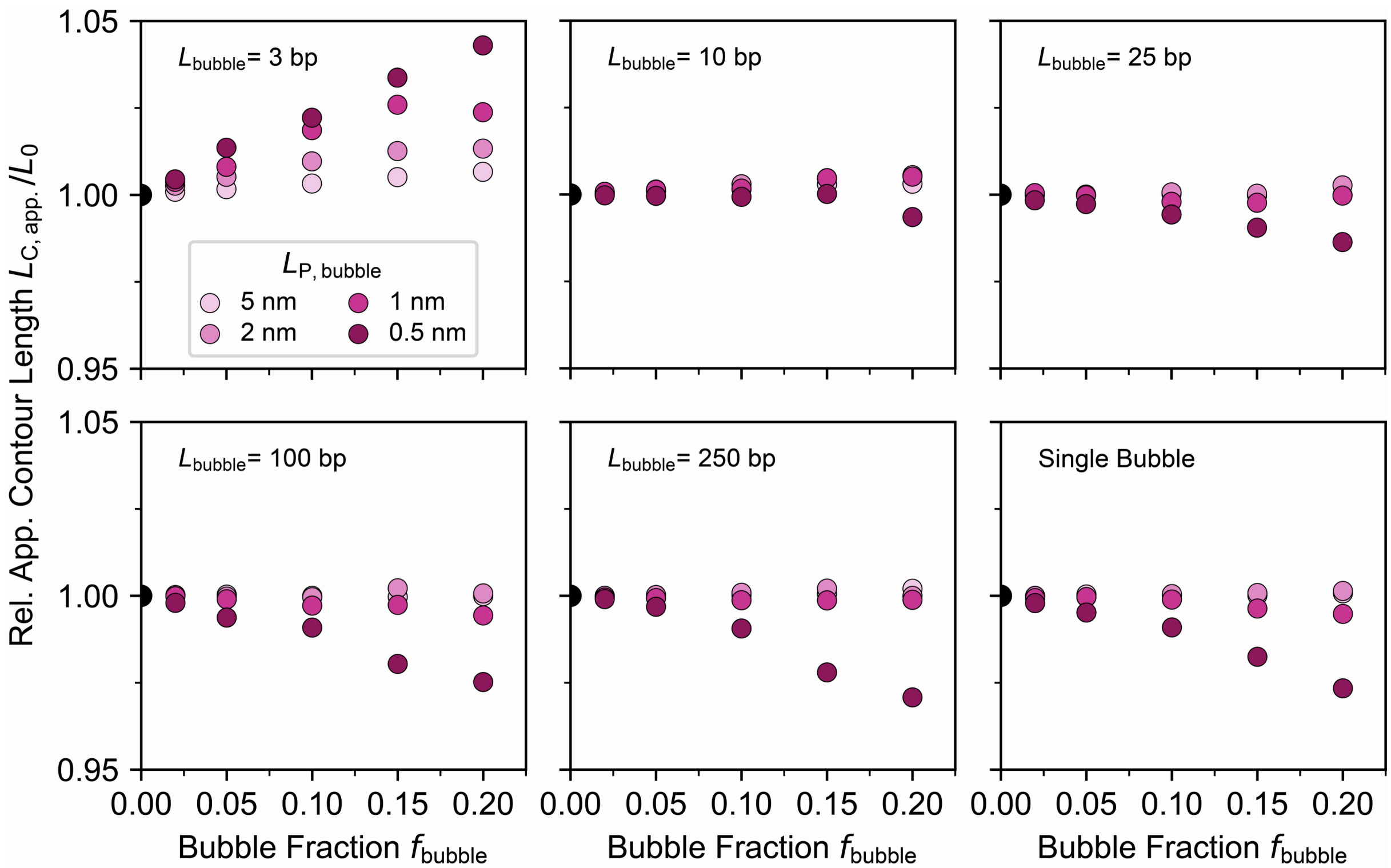

Supplementary Figure S13. Fitted contour lengths from coarse-grained DNA simulations with bubbles. Values for the fitted contour lengths obtained from WLC fits to the simulated force-extension responses of DNA with flexible bubbles (examples of the data and fits are shown in Supplementary Figure S10), normalized to the contour length of the chain in the absence of bubbles. Normalized values for the fitted apparent L_C are shown as a function of the bubble fraction and stiffness of the bubbles $L_{P,bubble}$ (color coded from dark to light shades; see legend in the top left panel; the same color code is used for all panels). The different panels show data for different bubble sizes L_{bubble}. The "Single Bubble" condition has L_{bubble} = 10,000 bp, such that all flexible bubble segments are in one cluster. Note the narrow scale of the y-axis. The fitted contour lengths are almost constant across the different conditions.

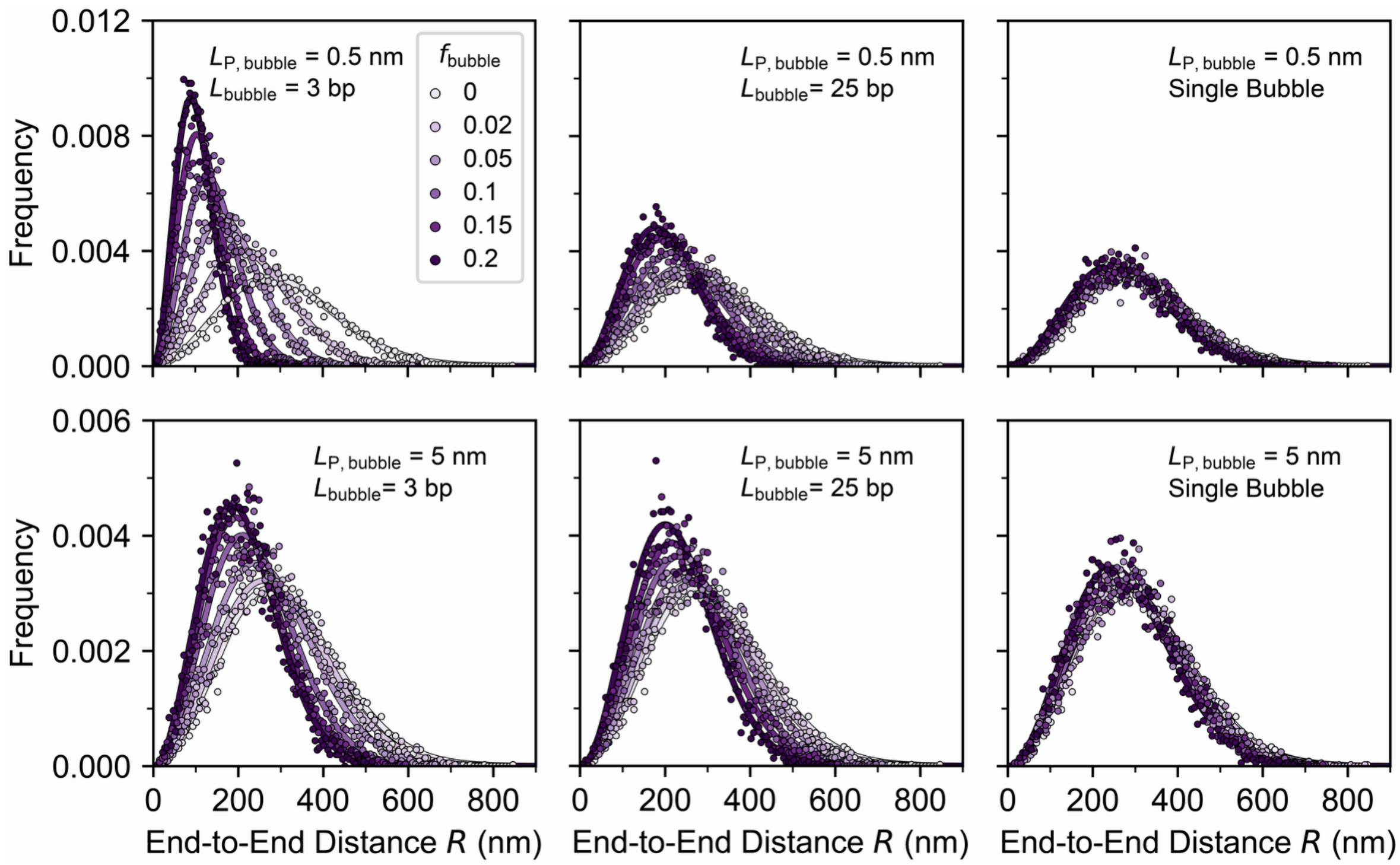

Supplementary Figure S14. End-to-end distance distributions for coarse-grained DNA simulations with bubbles. Distributions of end-to-end distances from coarse-grained Monte Carlo simulations (symbols) and fits of the Gaussian chain model (Equation 1; solid lines). The different colors in each panel indicate different bubble fractions f_{bubble} (increasing from light to dark shades; see legend in the top left panel; the same color code applies to all panels). The simulations systematically varied the flexibility of the bubble segments $L_{\text{P,bubble}}$ in the range 0.5 nm to 5 nm. Shown here are the data for $L_{\text{P,bubble}}$ = 0.5 nm (top row) and 5 nm (bottom row). In addition, we systematically varied the size of the flexible bubble regions, L_{bubble}, in the range of 3 bp to 10,000 bp. The 10,000 bp case corresponds to the situation that all bubble segments are in one cluster, i.e. that there is a single bubble that comprises all flexible segments. Shown are the data for L_{bubble} = 3 bp (left panels), 25 bp (middle panels), and the single bubble case (right panels).

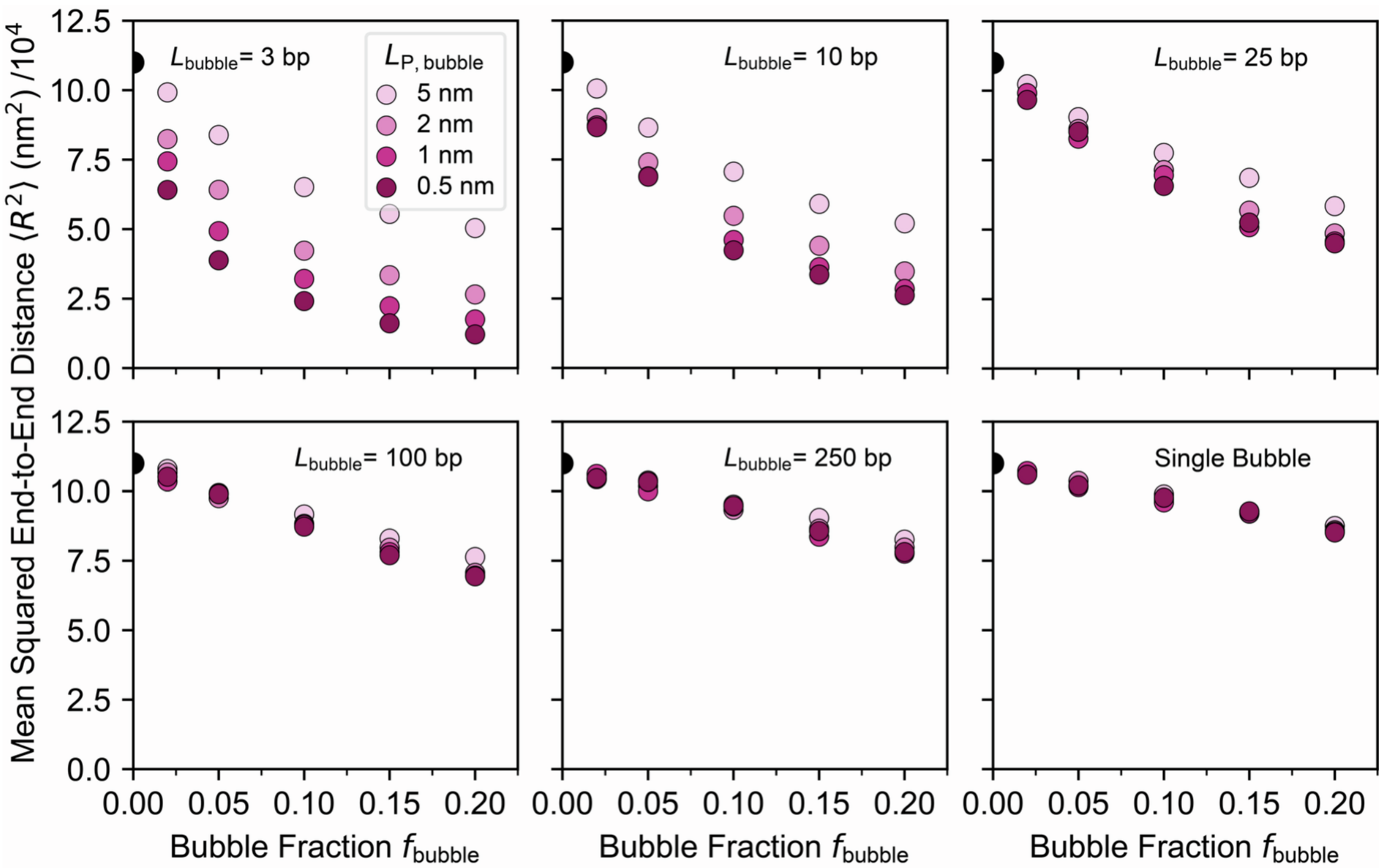

Supplementary Figure S15. Mean-squared end-to-end distance from coarse-grained DNA simulations with bubbles. Values for the mean-squared end-to-end distance $<R^2>$ obtained from the Monte Carlo simulations of DNA with flexible bubbles (examples of the distributions are shown in Supplementary Figure S13). Values for $<R^2>$ are shown as a function of the bubble fraction and stiffness of the bubbles $L_{P,bubble}$ (color coded from dark to light shades; see legend in the top left panel; the same color code is used for all panels). The different panels show data for different bubble sizes L_{bubble}. The "Single Bubble" condition has L_{bubble} = 10,000 bp, such that all flexible bubble segments are in one cluster.

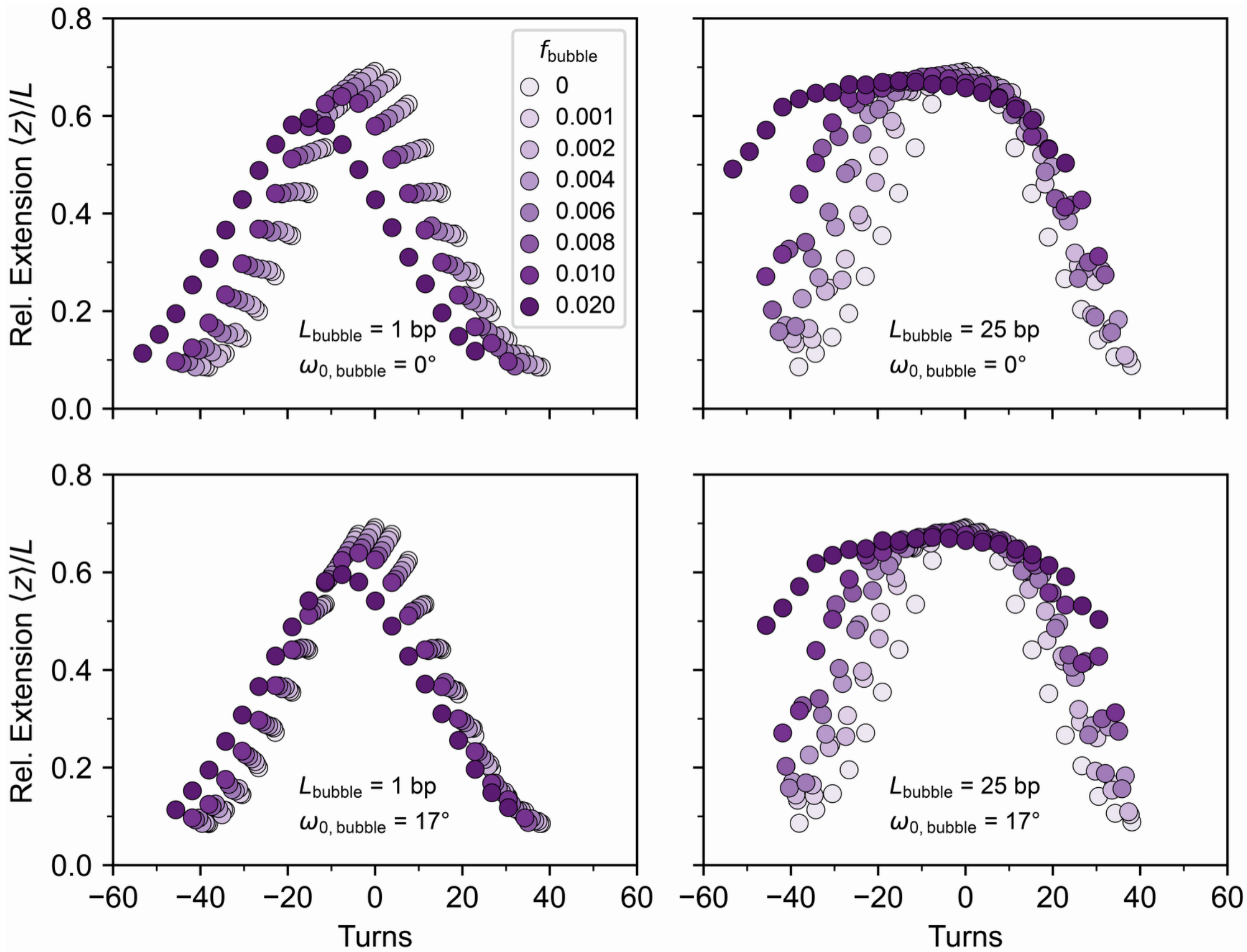

Supplementary Figure S16. Monte Carlo simulations of rotation-extension curves for DNA molecules with bubbles of different sizes. Rotation-extension simulations were performed for 8 kbp molecules subjected to a stretching force of $F = 0.25$ pN. Two bubble sizes were considered: 1 bp (left column) and 25 bp (right column). Additionally, two scenarios for the intrinsic twist in bubble steps were examined: one in which intrinsic twist is completely removed (top row) and one in which it is halved (bottom row). These correspond to a twist change of −34° and −17° per bubble step, respectively. Extensions are normalized to the contour length of the molecule. For small, dispersed bubbles, the rotation-extension curves remain largely unchanged, particularly at low bubble fractions, consistent with experimental observations. In contrast, large bubbles lead to fundamentally different behavior. The pre-buckling regime (the upper part of the curve, preceding the region of linear extension reduction) is significantly extended even at very low bubble fractions f_{bubble}. This effect arises because extended flexible regions (large bubbles) can efficiently absorb linking strain through local helicoidal or superhelical writhing. Consequently, these regions act as reservoirs for linking strain, which must be exhausted before the buckling transition of the remaining duplex DNA is initiated. These simulations further reinforce our conclusion that the modulation of DNA elasticity due to DMSO interactions is best explained by small, dispersed bubbles.
